# Metabolic reprogramming in cancer: Mechanisms and therapeutics

**DOI:** 10.1002/mco2.218

**Published:** 2023-03-27

**Authors:** Shiqi Nong, Xiaoyue Han, Yu Xiang, Yuran Qian, Yuhao Wei, Tingyue Zhang, Keyue Tian, Kai Shen, Jing Yang, Xuelei Ma

**Affiliations:** ^1^ State Key Laboratory of Oral Diseases West China Hospital of Stomatology West China School of Stomatology National Clinical Research Center for Oral Diseases Sichuan University Chengdu Sichuan China; ^2^ Department of Biotherapy Cancer Center West China Hospital Sichuan University Chengdu Sichuan China; ^3^ Department of Clinical Medicine West China School of Medicine West China Hospital Sichuan University Chengdu Sichuan China; ^4^ Department of Oncology First Affiliated Hospital of Nanjing Medical University Nanjing China; ^5^ State Key Laboratory of Oncology in South China Collaborative Innovation Center for Cancer Medicine Sun Yat‐sen University Cancer Center Guangzhou China; ^6^ Department of Biotherapy and Cancer Center State Key Laboratory of Biotherapy Cancer Center West China Hospital Sichuan University Chengdu Sichuan China

**Keywords:** cancer metabolism, cancer therapy, glycolysis, metabolic reprogramming

## Abstract

Cancer cells characterized by uncontrolled growth and proliferation require altered metabolic processes to maintain this characteristic. Metabolic reprogramming is a process mediated by various factors, including oncogenes, tumor suppressor genes, changes in growth factors, and tumor–host cell interactions, which help to meet the needs of cancer cell anabolism and promote tumor development. Metabolic reprogramming in tumor cells is dynamically variable, depending on the tumor type and microenvironment, and reprogramming involves multiple metabolic pathways. These metabolic pathways have complex mechanisms and involve the coordination of various signaling molecules, proteins, and enzymes, which increases the resistance of tumor cells to traditional antitumor therapies. With the development of cancer therapies, metabolic reprogramming has been recognized as a new therapeutic target for metabolic changes in tumor cells. Therefore, understanding how multiple metabolic pathways in cancer cells change can provide a reference for the development of new therapies for tumor treatment. Here, we systemically reviewed the metabolic changes and their alteration factors, together with the current tumor regulation treatments and other possible treatments that are still under investigation. Continuous efforts are needed to further explore the mechanism of cancer metabolism reprogramming and corresponding metabolic treatments.

## Graphical abstract

This article presented the metabolic reprogramming of tumor cells to adapt to their microenvironment distinct from normal cells. And this study reviewed the driving factors of metabolic reprogramming, the specific metabolic pathway changes affected, and the related treatment options, in order to provide some suggestions for future tumor treatment.

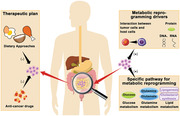

## 1 INTRODUCTION

Cancer is a group of complex genetic diseases characterized by autonomic and uncontrollable abnormal cell growth, which is mainly caused by complex changes in the genome involving mutations in oncogenes and tumor suppressor genes. The related changes promote the transformation of normal cells into malignant phenotypes. The microenvironment of malignant cells is different from that of normal cells. To adapt to hypoxia and nutritional deficiency, tumor cells must reprogram metabolic pathways to fulfill the energy, biosynthesis and redox needs of tumor cells.^1^ The “Warburg effect” in the process of tumorigenesis is currently one of the most widely studied reprogramming modes of cell metabolism. Even when there is enough oxygen, tumor cells still preferentially use glycolysis to produce adenosine triphosphate (ATP), and when the concentration of external nutrients and stress conditions are different, tumor cells will change their exact metabolic mode.^2^, ^3^ The occurrence of metabolic reprogramming can be driven by gene and protein levels and interactions between tumor cells and host cells, causing changes such as increased absorption of glucose in cancer. It is widely distributed in multiple metabolic pathways, including glucose metabolism, lipid metabolism, and amino acid metabolism. Tumor cells provide ATP through aerobic glycolysis, the pentose phosphate pathway (PPP), glutaminase (GLS) decomposition, one‐carbon metabolism, and de novo fatty acid synthesis, which produce a series of biological macromolecules to promote proliferation. The reprogramming of these metabolic pathways involves complex mechanisms and the coordinated interaction of various signaling molecules, proteins, and enzymes, such as mammalian target of rapamycin (mTOR) and adenosine monophosphate‐activated protein kinase (AMPK).

At present, tumor therapy for metabolism regulation can be divided into medication therapy and nutrition intervention therapy according to different intervention methods. Many drugs targeting metabolic reprogramming have been studied and are expected to become emerging strategies of metabolic regulation therapy for tumors aimed at metabolic complexity by acting on key proteins in related signaling pathways. Nutritional intervention is considered a traditional adjuvant treatment strategy for tumors. It changes the utilization rate of nutrients in the body cell microenvironment, including tumor cells, through diet regulation, thereby regulating many physiological processes of tumor cells. At the same time, an increasing number of studies have indicated that the combination of multiple strategies is also considered to be a promising treatment.

This paper reviews the growth characteristics of tumor cells and analyzes the metabolic mechanisms to adapt to the special growth needs of tumor cells from three aspects, glucose metabolism, lipid metabolism, and amino acid metabolism, and summarizes the driving factors of tumor metabolism reprogramming, the specific conditions of the changes in the three metabolic pathways, and the treatment targets available in metabolism reprogramming. Notably, this paper summarizes the factors driving reprogramming from the perspective of gene, protein and cell‒cell relationships and comprehensively summarizes the detailed drugs for each metabolic pathway target based on tumor metabolism reprogramming for the first time, which provides a promising direction for future tumor treatment.

## 2 DRIVERS OF METABOLIC REPROGRAMMING: RELATED GENES, PROTEINS, AND CELL–CELL INTERACTIONS

The tumor microenvironment (TME) is an internal environment with low oxygen content, acidic content and high permeability that tumor cells rely on for survival.^4^ To adapt to hypoxia and nutrient deficiency, tumor cells reprogram nutrient acquisition to fulfill the bioenergy, biosynthesis, and redox needs of tumor cells. This phenomenon is called metabolic reprogramming, which is a significant marker of cancer. It is widely observed in almost all tumorigenesis and development processes and is highly related to the transformation of cells, invasion, and metastasis of tumors. In recent years, metabolic reprogramming‐mediated microenvironment immunosuppression has been the bottleneck that causes immune escape and improves the efficacy of restricted immunotherapy.^5^ The specific mechanisms include hypoxia and nutrient deprivation in the TME leading to the establishment of metabolic competition between tumor cells and immune cells, as well as the continuous accumulation of toxic metabolites, which will have a negative impact on the immune response. On the other hand, high metabolism and strong metabolic adaptability of tumor cells further change the metabolic characteristics of the TME, exert metabolic pressure on infiltrated immune cells, and promote immunosuppression and immune escape.

### 2.1 Alterations in genes

Oncogenes and tumor suppressor genes can affect the glucose metabolism of tumor cells through a variety of mechanisms, which are the primary factors that cause tumorigenesis and drive reprogramming of glucose metabolism. Activation of oncogenes leads to metabolic reprogramming, while tumor suppressor genes lead to reprogramming through mutations or deletions. Tumor suppressor genes such as p53 have a negative regulatory effect on cell metabolism changes caused by excessive activation of oncogenes and inhibit cell biosynthesis and metabolism and energy supply pathways.

#### 2.1.1 Oncogenes

##### 2.1.1.1 Cellular myelocytomatosis oncogene (c‐MYC)

The cellular myelocytomatosis oncogene (c‐MYC) is related to tumor development by participating in metabolism, cell cycle regulation, cell adhesion, protein synthesis, cytoskeleton, apoptosis, and angiogenesis, and its expression in normal cells is strictly controlled, but it is highly expressed in most tumors. As a transcription factor, c‐MYC promotes many cells proliferation‐related target genes, such as cyclin‐dependent kinase 4 (CDK4) and RNA polymerase III. Therefore, upregulation of MYC expression in cells will contribute to cell hyperproliferation.^6^ MYC positively affects many regulatory processes, such as tumor cell adhesion, antiapoptosis, and tumor angiogenesis, and its expression level in tumor tissues is closely related to tumor progression and prognosis. Abnormal activation of c‐MYC due to chromosomal translocation, gene amplification, or transcriptional enhancement is significant in the occurrence and progression of various tumors and is closely related to the occurrence of metabolic reprogramming.^7^

In carcinogenesis, MYC enhances glycolysis by regulating the expression of glycolysis genes and related metabolic enzymes, including glucose transporter 1 (GLUT1),^8^, ^9^ l‐lactate dehydrogenase A (LDHA), hexokinase (HK) 2, phosphofructokinase (PFK), muscle (PFKM), pyruvate dehydrogenase kinase 1 (PDK1), and α‐enolase. In addition, MYC can directly activate the gene transcription of monocarboxylate transporter 1/2 (MCT1/MCT2) or by inhibiting miRNA (e.g., Mir‐29a and Mir‐29c).^10^, ^11^ MYC can also regulate the PPP by promoting aerobic glycolysis, which can increase the production of ribose and nicotinamide adenine dinucleotide (NAD) phosphate (NADPH). In addition, MYC plays an important role in regulating glutamine transportation and catabolism in tumor cells. It supports the functional tricarboxylic acid (TCA) cycle by enhancing the expression of glutamine synthase, GLS, and alanine‐serine‐cysteine transporter 2 (ASCT2). The substrate for anabolic pathways is ensured by increased glycolytic flux and glutaminolysis. Regarding lipid metabolism, MYC plays an essential role by regulating the key enzymes for fatty acid de novo synthase, such as acetyl‐CoA carboxylase (ACC), fatty acid synthase (FASN), stearoyl‐CoA desaturase (SCD), and so on. MYC can also promote fatty acid oxidation (FAO).

In conclusion, MYC plays a significant role in the biological process of tumor cells by promoting glycolysis, glutamine decomposition, nucleotide synthesis, fatty acid metabolism and other pathways to promote the proliferation of tumor cells.

##### 2.1.1.2 Kirsten‐ras oncogene

As one of the most frequently mutated oncogenes in cancer, approximately 30% of all human cancers have Kirsten‐ras oncogene (KRAS) mutations, which have extensive effects on tumor transformation. Recent studies have indicated that KRAS plays a key role in coordinating tumor metabolic reprogramming by upregulating the transcription of multiple key glycolysis enzymes, such as HK1/2, PFK1, LDHA, and GLUT1. It is also responsible for reprogramming various aspects of central carbon metabolism. These metabolic changes are crucial for the growth of KRAS‐mutated tumors.

KRAS mutation can increase glucose uptake by regulating glucose transporters and glycolytic enzymes,^12^, ^13^ which can enhance the Warburg effect in tumor cells. At the same time, it can also lead to the diversion of intermediate metabolites of glycolysis into specific anabolic pathways. KRAS upregulates the expression of the transcription factor MYC through activation of the MEK/extracellular regulated protein kinases (ERK) pathway to enhance glycolytic metabolism. In addition, KRAS enhances the hexosamine biosynthesis pathway (HBP) and produces the precursor substances required for glycosylation, which plays a key role in tumorigenesis as an important posttranslational modification process.^14^ In addition, KRAS mutation promotes ribose biosynthesis by diverting glycolytic intermediates into the nonoxidation arm of the PPP.^12^ With the upregulation of ribulose‐5‐phosphate‐3‐epimerase and ribose‐5‐phosphate (R5P) isomerase A (RPIA), an increase in PPP flux can be observed. Meanwhile, KRAS participates in glutamine metabolism via an alternative pathway, which is through the regulation of glutamic‐oxaloacetic transaminase, malate dehydrogenase 1 (MDH1) and malice enzyme 1 (ME1). Thus, KRAS can affect the biosynthesis of reduced NADPH, which is closely linked to redox balance and tumor growth.^15^, ^16^ Lipid metabolism, especially fatty acid synthesis, is a biological process^14^ necessary for membrane biosynthesis, signal molecule production, and energy storage. KRAS mutation can regulate β‐oxidation of fatty acids in non‐small cell lung cancer (NSCLC) and de novo synthesis processes to participate in tumor metabolic reprogramming and regulate fatty acid metabolic enzymes to regulate fatty acid production in lung cancer cells.^17^, ^18^ The KRAS mutation may also have a synergistic effect with mutations in the signal transduction GNAS gene that promote the growth of pancreatic ductal adenocarcinoma (PDAC) by inducing salt‐inducible kinases.^19^

##### 2.1.1.3 AK mouse plus transforming or thymoma

The AK mouse plus transforming or thymoma (AKT) oncogene can encode a Ser/Thr‐specific protein kinase that mediates the PI3K signaling pathway. The upstream signaling pathway, the epidermal growth factor receptor (EGFR) signaling pathway, participates in the control of tumor cell proliferation, inhibits cell apoptosis, and promotes cell cycle progression. Constitutive activation of the PI3K/AKT signaling pathway by AKT1 occurs in various cancers. Gene amplifications or activation mutations of the AKT gene lead to its overactivation and transformation of tumor cells. Growth factor receptor tyrosine kinase, PI3K, or Ras can also promote AKT activity. Activation of AKT is likely to contribute to the elevation of glycolysis metabolism in cancer cells.

##### 2.1.1.4 Isocitrate dehydrogenase 1/2

Mutations in isocitrate dehydrogenase (IDH)1/2 alter enzyme activity and result in the conversion of protein from α‐KG to 2‐hydroxyglutarate (2‐HG). 2‐HG isomers are structurally similar to α‐KG, which can competitively inhibit the α‐KG‐dependent dioxygenase family, such as 5‐methylcytosine. IDH1/2 participates in changes in tumor metabolism through glutamine metabolism reprogramming.^20^ Thus, the epigenetic state of cells is changed, and the normal biological behavior of cells is affected. It can also reduce the expression level of hypoxia‐inducible factor (HIF) and promote the proliferation and growth of tumors.

##### 2.1.1.5 Phosphatidylinositol 3‐kinase

The phosphatidylinositol 3‐kinase (PIK3CA) gene is located on chromosome 3 and belongs to the PI3K/AKT signaling pathway. It is responsible for encoding protein P110α, which is one of the catalytic subunits of the PI3K enzyme. Mutation of PIK3CA can activate PI3K continuously and enhance intracellular signal transduction, which leads to disorder of the whole pathway and carcinogenesis.

##### 2.1.1.6 Epidermal growth factor receptor

Mutations of the EGFR change its structure and remain active constantly. It plays an important role in tumorigenesis by regulating cell metabolism and inducing glycolysis, PPP, pyrimidine biosynthesis, and redox metabolism. Mutated EGFR reprograms the metabolism of tumor cells through the PI3K/AKT/mTOR pathway.^21^

##### 2.1.1.7 Octamer‐binding transcription factor

Octamer‐binding transcription factor (Oct4), an oncogene, plays significant roles during cancer initiation, progression, drug resistance, and relapse. It enhances the expression levels of Oct4 metabolic programming by reprogramming differentiated cells into cancer stem cells and plays an oncogenic role when pathologically hijacked. Some studies have identified that overexpressed Oct4 redirects glucose catabolism to the glycolysis pathway and to the oxidative PPP. Nevertheless, there are limited data on Oct4‐induced metabolic reprogramming and a lack of direct evidence to prove that Oct4 reprograms the metabolome at all metabolic levels.

#### 2.1.2 Tumor suppressor genes

Apart from the overactivation of oncogenes, the inactivation of tumor suppressor genes is also an important initiating factor of tumor metabolic reprogramming. The tumor suppressor genes p53, liver kinase B1 (LKB1), and tuberous sclerosis complex 2 (TSC2) have negative regulatory effects on cell metabolism changes and inhibit cell bio‐anabolism and energy supply pathways.

##### 2.1.2.1 p53

p53, the most frequently mutated gene in human cancer, regulates the transcription of many target genes, which can not only promote DNA damage repair and cell survival but also promote apoptosis and remove unrepairable cells, thereby maintaining genomic integrity and playing a role in inhibiting the occurrence and development of tumors. It plays an important role in glucose metabolism, lipid metabolism, and oxidative phosphorylation (OXPHOS). However, mutations or deletions can lead to the loss of functions, thus promoting metabolic reprogramming. The specific mechanism of p53 during reprogramming to inhibit tumor metabolism is as follows.

Under physiological conditions, p53 can directly inhibit the gene expression^22^ of GLUT1 and GLUT4 or inhibit the expression of GLUT3^22^ through the nuclear factor‐κB (NF‐κB) signaling pathway,^23^ thus reducing glucose intake and the flux of glycolysis metabolism. p53 also activates TIGAR gene expression^24^ through transcription and reduces intracellular fructose 2,6‐diphosphate content through a series of metabolic processes, thereby reducing the activity of fructose‐6‐phosphate kinase‐1, the rate‐limiting enzyme of glycolysis, to inhibit glycolysis. In addition, p53 activates ubiquitination of phosphoglucomutase,^25^ which promotes phosphoglycerate mutase (PGM) degradation and inhibits glycolysis. Therefore, inactivation of p53 accelerates glycolysis in tumor cells. In addition, p53 also inhibits lactate transport by inhibiting MCT1 and leading to lactic acid accumulation under hypoxic conditions. In addition, p53 can regulate HK2, cytochrome *C* oxidative synthase 2 and other enzymes to inhibit tumor development. In the PPP, p53 inhibits its activity by binding with glucose‐6‐phosphate (G6P) dehydrogenase, thus inhibiting the PPP. It reduces the generation of 5‐phosphoribose and NADPH and the generation of raw materials for malignant tumor growth. For glutamine metabolism, p53 increased glutamate and α‐ketoglutaric acid contents, enhanced mitochondrial oxidative respiration,^26^ and increased ATP production through continuous transcriptional activation of GLS2. In addition, GLS2 reduces reactive oxygen species (ROS) levels by increasing glutathione (GSH) content and protects cells from oxidative stress damage. In conclusion, as a target gene of p53, GLS2 functions as a tumor suppressor gene by regulating energy metabolism and resisting oxidative stress.^26^, ^27^ It also lowers the levels of key glycine hydrolases such as HK2, PGM1, and PDK2. In cancer cells, mutated p53 induces the expression of genes related to lipid metabolism by binding to and activating sterol regulatory element binding protein (SREBP), thus promoting lipid metabolism in various tumors. In normal cells, p53 induces malonyl‐CoA decarboxylase, which catalyzes the conversion of malonyl‐CoA to acetyl‐CoA, Lipin1, and SIRT, preventing lipid accumulation.

In conclusion, p53 is closely related to tumor cell metabolism. P53 can inhibit tumors by regulating the expression or activity of related metabolic enzymes, inhibiting aerobic glycolysis of glucose, inhibiting PPP, enhancing OXPHOS of glucose, and inhibiting de novo fatty acid synthesis. Mutation of p53 leads to a decline in all its activities. The enhancement of glycolysis flux, PPP, lipid metabolism, and declination of mitochondrial respiratory activity through loss of synthesis of cytochrome *C* oxidase‐2 promote the occurrence and development of tumors.

##### 2.1.2.2 Phosphatase and tensin homolog deleted on chromosome ten

As a cancer suppressor gene, phosphatase and tensin homolog deleted on chromosome ten (PTEN) has dual specificity phosphatase activity and inhibits cancer through apoptosis, cell cycle arrest, cell migration, and so on. The balance of the PI3K/AKT signaling pathway is the key to regulating these functions.^28^ Metabolic reprogramming resulting from mutations or deletions of PTEN induces the growth and proliferation of cancer cells by altering intracellular metabolism.^28^ The coding products of the PTEN gene can induce the CDK inhibitors P21, P27, and P51 to participate in cell cycle regulation or promote GLUT1 expression by antagonizing the AKT signaling pathway to change the apoptosis of cells. In addition, the PTEN protein plays an important role in the occurrence and development of tumors by inhibiting cell adhesion and invasion, inhibiting neovascularization and regulating drug resistance.

##### 2.1.2.3 Liver kinase B1

LKB1, a serine/threonine kinase, can regulate cell polarity, energy metabolism, proliferation, and migration. Mutations and disorders of LKB1 can be observed in a variety of cancers.^29^ Research has shown that the loss of LKB1 can trigger complex changes in the TME, which promote the formation of blood vessels and an immunosuppressive microenvironment and the occurrence and development of cancer. Therefore, LKB1 plays a vital role in the pathogenesis of tumors.

Deletion of LKB1 can plunge cells into an energy/oxidative stress‐induced crisis, which contributes to the activation of carcinogenic pathways so that the cell energy levels can be maintained. Silencing LKB1 can reduce the phosphorylation of AMPK, thereby increasing the phosphorylation of protein kinase B and promoting tumor cell proliferation. Thus, the migration and invasion of colon cancer cells are enhanced. In addition, the increase in LKB1 promoter methylation frequency is another major mechanism of LKB1 inactivation. Moreover, LKB1 can regulate AMPK (a central metabolic sensor that controls glucose and lipid metabolism, facilitating the transition from anabolism to catabolism when energy is lacking) to transduce the signal to promote metabolism in response to energy metabolic stress. Therefore, deletion of LKB1 in tumor cells is sensitive to energy metabolic stress. In addition, deletion of LKB1 can lead to metabolic changes in HIF‐1‐dependent proliferating cells. HIF‐1 accumulates in LKB1‐deficient cells and leads to increased absorption and utilization of glucose and glutamine,^30^ thus promoting the occurrence and development of tumors.

##### 2.1.2.4 Tuberous sclerosis complex 2

TSC2 functions as a negative regulator of mTOR signaling. When the TSC2 gene is mutated, the mTOR complex 1 (mTORC1) signal transduction pathway is abnormally activated.^31^ The PI3K/AKT/mTOR pathway plays an important role in tumorigenesis, and mTOR is a key regulator in this process.^32^ Therefore, enhanced activation of mTOR is important for metabolic reprogramming, proliferation, and survival.

#### 2.1.3 RNA

In addition to oncogenes and tumor suppressor genes, noncoding RNAs (ncRNAs) are important participants in the reprogramming mechanisms of cancer metabolism. Although ncRNAs were initially thought to lack biological function due to their inability to encode proteins, they can play a role in cancer progression by regulating enzymes and pathways involved in cancer cell metabolic reprogramming. This regulation occurs mainly through glucose, glutamine, and lipid metabolism and involves two types of ncRNA,^33^ long noncoding RNA (lncRNA) and microRNA (miRNA).

##### 2.1.3.1 Long noncoding RNA

lncRNAs are a heterogeneous group of nonprotein coding transcripts with a length greater than 200 nucleotides. lncRNAs are newly discovered regulatory factors involved in regulating gene expression and a variety of physiological and pathological processes. Studies have shown that lncRNAs play a complex and precise regulatory role in the occurrence and development of cancer by exerting oncogene or tumor suppressor gene activity or sometimes functioning as these genes.^34^ They not only regulate the proliferation, differentiation, invasion, and metastasis of cancer cells but also regulate metabolic reprogramming. In regulating the transcription and translation of metabolism‐related genes, lncRNAs play important roles by acting as bait molecules, scaffold molecules, and competing endogenous RNAs (ceRNAs), which ultimately lead to cancer metabolic reprogramming.^35^ lncRNAs promote energy metabolism and cancer progression through posttranslational modifications of key metabolite‐related proteins, and they can activate glycolytic flux by binding 6‐phosphofructo‐2‐kinase/PFK2/fructose 2,6‐bisphosphatase (PFKFB3).

##### 2.1.3.2 MicroRNA

miRNAs are a class of 22 bp small single‐stranded ncRNAs that regulate gene expression at the posttranscriptional level and are widely involved in various physiological and pathological processes. The abnormal expression of miRNA in tumor development is closely related to tumor progression. In cancer, miRNAs regulate enzymes associated with glucose metabolism. For example, HK2 expression is downregulated by miR‐199a‐5p and miR‐125b, and low expression of these ncRNAs corresponds to the enhancement of tumor growth.^33^ In addition, in breast cancer, the secretion of miR‐122 vesicles can downregulate GLUT1 and pyruvate kinase (PK) expression and reduce glucose uptake in nontumor cells, thereby increasing premetastatic cell nutrient availability and promoting metastasis.^36^ Glutamine metabolism and lipid metabolism reprogramming are also affected by ncRNAs. Although most of the ncRNAs involved in these metabolic pathways inhibit tumor metastasis, a portion of ncRNAs can also promote tumor growth. ncRNA mainly regulates the PI3K/AKT/mTOR pathway. For example, miR‐149‐5p can activate this pathway to promote tumor cell growth, and this pathway can also be activated by other factors, such as miR‐384^33^ after upregulation of multitrophic factors and lipogenic genes. ncRNA, which plays an important role in affecting metabolic processes and activating carcinogenic signaling pathways, is gradually receiving attention.

### 2.2 Protein

#### 2.2.1 Transforming growth factor β

Of the various growth factors, transforming growth factor β (TGF‐β), which controls growth and differentiation, plays an important role in carcinogenesis. Under physiological conditions, TGF‐β is involved in the regulation of some important cellular functions, such as differentiation, amplification, migration, and immune monitoring.^37^ An imbalance in the regulation of this growth factor may lead to developmental abnormalities, immune‐related diseases, tumorigenesis, and so on.^37^ TGF‐β plays a role in promoting angiogenesis and immune escape in tumors and can induce the process of epithelial–mesenchymal transition (EMT), which plays an important role in increasing the invasiveness of tumor cells and tumor survival. The TGF‐β and PI3K/AKT pathways can activate mTOR in tumor cells, regulate endothelial cell growth, and enhance the characteristics of tumor stem cells.^38^ Activation of the mTOR and PI3K pathways can resist tumor cell apoptosis and induce the EMT process.^39^ Therefore, high expression of mTOR is related to poor prognosis in some types of tumors and needs to be considered. In addition, TGF‐β induces the necessary glycolytic enzymes to increase the glycolytic flux.^40^ TGF‐β participates in the regulation of lipid metabolism through the protein kinase C (PKC)‐dependent fatty acid transporter CD36 (CD26/FAT), which facilitates the absorption of long‐chain fatty acids in the mitochondria for subsequent oxidation and productivity.^41^

#### 2.2.2 Hypoxia‐inducible factor

The hypoxic microenvironment is an important feature of solid tumor tissue and has an important influence on tumor extracellular matrix structure, immune response, angiogenesis, metastasis and other processes. Hyperglycolysis and reduced OXPHOS are the main adaptive changes in tumor metabolic reprogramming induced by hypoxia. HIF‐1 is an oxygen‐sensitive transcription factor and is one of the main regulatory factors of tumor metabolic reprogramming that promotes hypoxia‐induced gene expression and induces a specific response of the body tissue to hypoxia. It is highly expressed in many tumors, such as colorectal cancer,^42^ pancreatic cancer,^43^ and breast cancer,^44^ and is closely linked to the glycolytic pathway of tumor cells. HIF‐1 can enhance glycolysis in cancer cells by upregulating the expression of GLUT1 and LDHA in breast cancer cells. In other cancers, HIF‐1 can affect tumor growth by regulating various enzymes of tumor glycolysis. Moreover, HIF‐1 and c‐MYC can work together to promote the expression of PDK1 and HK2 and jointly promote glycolysis. In addition, HIF‐1 can also affect the TCA cycle of tumor cells by regulating various metabolic enzymes. Thus, HIF‐1 can affect tumor metabolic reprogramming by regulating multiple key enzymes of tumor glycolysis. In addition to glycolysis, HIF‐1 also has an effect on tumor lipid metabolism. In gastric cancer,^45^the high expression of HIF‐1a is related to poor prognosis, and the main mechanism is that HIF‐1α can upregulate FASN activity and then activate SREBP‐1C. Inhibition of FASN can reduce the expression, activity, and ubiquitination of HIF‐1α, thus inhibiting the migration and invasion of HCC cells.^46^ In conclusion, HIF‐1 participates in multiple processes of tumor metabolism and plays a vital role in the process of tumor cell proliferation.

Other growth factors with less research are also involved in tumor processes. For example, epidermal growth factor (EGF) promotes the EMT process through metabolic reprogramming and promotes PDK1 expression through the EGFR/PI3K/HIF‐1 axis, thus promoting glycolysis. EGF also promotes lipid synthesis by providing acetyl‐CoA with glutamine and regulates the synthesis of monounsaturated fatty acids by phosphorylation of SCD1.^47^, ^48^

#### 2.2.3 Forkhead box

Forkhead box (Fox) proteins are a large family of transcription factors that play an important role in various biological processes. Based on the similarity of genes in the Fox family, Fox proteins can be divided into 19 subfamilies, from FoxA to FoxS. Among them, the FoxO and FoxM subfamilies are most closely related to tumor metabolism. The FoxO transcription factor family controls the occurrence and development of tumors by influencing tumor metabolism, such as glucose metabolism, glutamine metabolism and lipid metabolism. In terms of glucose metabolism, FoxO inhibits glycolysis gene expression, glucose uptake, and lactic acid production by regulating MYC and affects the Warburg effect.^49^ Regarding glutamine metabolism, FoxO3 and FoxO4 can regulate the expression of glutamine synthetase and directly affect the metabolism of glutamine. FoxO3 can lead to autophagosome formation by increasing the activity of glutamine synthase and contribute to FoxO3 inhibiting the proliferation of tumor cells. In addition, FoxO1 can promote lipolysis by upregulating the expression of fatty triglyceride lipase.^50^ FoxM1 is indispensable for normal cell proliferation, highly expressed in many cancers^51^, ^52^, ^53^ and closely associated with poor prognosis. One of the mechanisms by which FoxM1 promotes tumor development is the reprogramming process of cell metabolism. FoxM1 promotes glycolysis, proliferation, and migration of cancer cells through transcriptional regulation of LDHA. In addition, FoxM1D promotes tumor angiogenesis by binding to pyruvate kinase M (PKM) 2, thereby promoting glycolysis and enhancing vascular endothelial growth factor (VEGF) transcription.^54^ At present, the effects of the Fox family on tumor metabolism mainly focus on glycolysis and lipid metabolism, while the effects of the Fox family on other metabolic pathways need more research.

#### 2.2.4 E2F transcription factor 1

E2F transcription factor 1 (E2F1) is a member of the cell cycle‐related transcription factor family. It not only affects cell cycle progression and cell proliferation through transcriptional regulation but is also closely related to metabolism.^55^, ^56^ It has been proven in experiments that E2F1 gene knockout can produce obvious metabolic disorders, which can be shown in the impairment of glucose homeostasis, mitochondrial function impairment and the reduction of pancreatic volume. E2F1 can inhibit glucose oxidative metabolism in a variety of ways and promote the transformation of tumor cell metabolism from OXPHOS to aerobic glycolysis. In addition to regulating the glycolysis of tumor cells, E2F1 can also change the lipid metabolism of cells by regulating key genes for lipid metabolism, such as Serpine mRNA binding protein 1 and peroxisome proliferator‐activated receptor γ,^57^ to provide the substances needed for cell membrane synthesis and provide the basis for the rapid proliferation of tumor cells.

#### 2.2.5 High mobility group box 1

High mobility group box 1 (HMGB1) is a nonhistone chromosomal binding protein existing in eukaryotic nuclei. HMGB1 regulates inflammatory pathways, immunity, genomic stability, cell proliferation, migration, apoptosis, and autophagy^58^ by binding to receptors of advanced glycation end products (RAGE) and some Toll‐like receptor families^59^ and is widely involved in the occurrence, growth, invasion and metastasis of tumors.

#### 2.2.6 mTOR complex 1

mTOR is a highly conserved serine/threonine protein kinase in the PI3K‐related kinase family that plays an important role in regulating cell proliferation and metabolism. mTORC1 regulates cellular metabolism from protein synthesis, lipid synthesis and mitochondrial activity by receiving upstream signals related to nutrients and hormones. mTORC2 promotes cell survival by activating AKT, regulates cytoskeletal dynamics by activating PKCα, and controls ion transport and growth through phosphorylation of serum and glucocorticoid‐regulated kinase 1 (SGK1).^60^ mTOR2 also participates in glutamine metabolism and promotes glutamine uptake by activating AGC kinase, which is named after protein kinase A, G, and C families (PKA, PKC, PKG). The mTOR signaling pathway plays an important role in tumor metabolic reprogramming.

#### 2.2.7 Insulin‐like growth factor receptor

The insulin‐like growth factor (IGF) signaling pathway is a complex network consisting of two ligands (IGF‐1 and IGF‐2), two receptors (IGF‐1R and IGF‐2R) and six IGF‐binding proteins (IGF‐BP1‐6). IGF‐1R is upregulated in most malignant tumors, and its combination with the ligands IGF‐1 or IGF‐2 can activate the PI3K/AKT and Ras/Raf/MEK/ERK/MAPK signaling pathways, thereby promoting tumor proliferation, differentiation, and migration and playing a role in fine‐cell apoptosis inhibition. On the one hand, the downstream signaling pathway of IGF‐1R has multiple crossing sites with oncogenes such as Ras and c‐MYC, which can regulate each other to play a role in leading to tumor formation. On the other hand, it can also interact with EGFR and vascular EGFR pathways to regulate cell proliferation and differentiation. Activation of IGF‐1R leads to upregulation of HIF 1α protein synthesis and thus leads to expression of VEGF, a core mediator of angiogenesis.^61^

### 2.3 Interaction between cells

Reprogramming of metabolic pathways in cancer is not only limited to the tumor cells themselves but also involves the interaction of tumor cells with the host cells themselves.^62^ It is related to a variety of cells in the TME, such as fibroblasts, macrophages, mesenchymal cells, and epithelial cells. The metabolic interaction between different cell populations is conducive to tumor growth and proliferation.

Nutrient recycling between tumor cells and fibroblasts is vital for tumor growth. Fibroblasts mainly produce and secrete extracellular matrix components, such as collagen, which can be absorbed by tumor cells for metabolism. Cancer‐associated fibroblasts (CAFs)^63^ are a major subpopulation of basal plasma cells in the TME and are also highly dependent on CAFs for tumor progression. CAFs can secrete various growth factors and chemokines that promote tumor growth and metastasis. Studies have shown that the metabolism of CAFs is reprogrammed to switch from OXPHOS to aerobic glycolysis and that CAFs and tumor cells dominate the microenvironment. Upon interaction with tumor cells, CAFs can mobilize glycogen as a source of energy, accelerating tumor progression and spread. The interaction between tumor cells and CAFs induces phosphorylation and activation of PGM1, which promotes the transport of glycogen to glycolysis, leading to increased proliferation, invasion, and metastasis of tumor cells.

In addition, competition between tumors and host cells can affect tumor growth. To obtain the energy needed for their growth and development, tumor cells, host cells, and stromal cells have a competitive relationship, and due to the weak competitiveness of immune cells,^62^ they lose their immune monitoring function, causing tumor growth. Glucose utilization is the key to glycolysis, and thus, glucose consumption in the TME observed in many cancer types can reduce the antitumor activity of immune cell populations and contribute to cancer progression.^64^ In addition, the metabolism of amino acids, lipids, and one carbon unit by cancer cells also affects the metabolism of immune cells.

Tumor cells undergo metabolic reprogramming to adapt to their physiological characteristics of low oxygen demand and rapid proliferation. There are many driving factors, but they can be roughly divided into three aspects: gene, protein, and cell. Specific changes in cellular metabolism are discussed in detail in the following sections.

## 3 METABOLIC CHANGES OF GLUCOSE, AMINO ACID, LIPID, AND OTHERS

### 3.1 Glucose metabolism: the most representative metabolic feature in tumors

**FIGURE 1:**
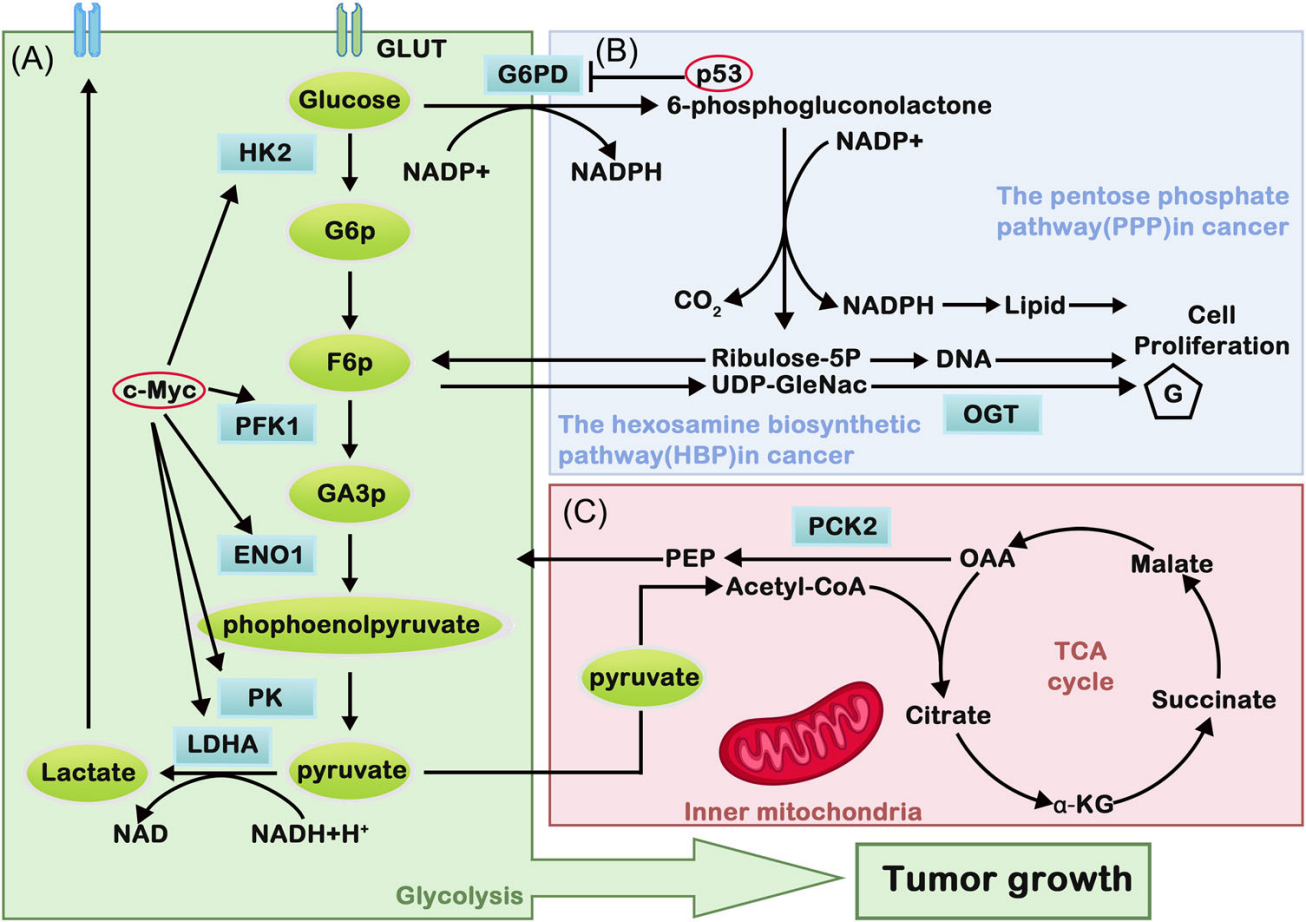
Glucose metabolic reprogramming in cancer. (A) Glycolytic pathway: c‐MYC can promote glycolysis by upregulating GLUT and several glycolytic enzymes. This increased process in tumor cells adapts to metabolic changes and promotes growth. (B) The pentose phosphate pathway (PPP) and the hexosamine biosynthetic pathway (HBP): the PPP is the main glucose catabolism pathway that links glucose metabolism with NADPH production. It provides NADPH and ribose‐5‐phosphate (R5P), which plays a key role in the elimination of cellular ROS and reductive biosynthesis. Meanwhile, p53 affects PPP by inhibiting the expression of G6PD. Therefore, it is always mutated in cancer. For HBP, UDP‐GlcNAc levels are increased by upregulating HBP enzymes. (C) The tricarboxylic acid cycle pathway (TCA): pyruvate, one of the intermediates in glycolysis, enters the mitochondria and participates in the TCA cycle.

Glucose metabolic reprogramming is one of the representative metabolic features in tumors. The mechanism of energy metabolism is different between tumor cells and normal cells, in which the former does not utilize mitochondrial OXPHOS capacity even under aerobic conditions, and the active aerobic glycolysis phenomenon is recognized as the Warburg effect.^65^, ^66^ Aerobic glycolysis is inefficient in obtaining energy when compared with OXPHOS. However, tumor cells still tend to use energy sources such as aerobic glycolysis. The reason behind this is that the intermediate metabolites produced by the high flux of aerobic glycolysis can promote nucleotide, amino acid, lipid, and other important macromolecular synthesis, which can fulfill the needs for tumor cells to proliferate rapidly.^67^ Nevertheless, the latest studies on the source of biosynthesis suggest that the increase in glucose and glutamine caused by the Warburg effect is not the main provider of substance accumulation.^68^ Therefore, the mechanism of how the Warburg effect affects tumor metabolism is still questionable and needs further research. In tumor cells, glucose metabolism includes the glycolysis pathway, the TCA, PPP, and HBP (Figure 1). These pathways can be recognized to provide ATP and glutamine, serine, arginine, fatty acids, and lipids rapidly to promote tumor self‐proliferation, which are known as glucose metabolic glycolytic pathways.

#### 3.1.1 Glycolysis pathway: the major pathway to gain ATP in tumors

In contrast to normal cells, approximately 50% of ATP is synthesized through the glycolysis pathway in tumor cells, which is why glycolysis can supply tumor cells with sufficient energy.^69^ The tolerance of tissue cells toward hypoxia is enhanced, together with avoiding apoptosis induced by OXPHOS. In addition, the glycolysis pathway can initiate autonomous nutrient uptake in tumor cells. Normal cells require exogenous stimulation signals to initiate nutrient uptake, while tumor cells can take nutrients directly through glycolysis.^67^ After glucose intake increases due to metabolic reprogramming, lactic acid is produced in large quantities, which provides tumor cells with energy and a microenvironment conducive to their own abnormal growth, proliferation and metastasis, avoiding tissue damage that may be caused by local glucose deficiency under hypoxia, thus promoting tumor cell growth.

The glycolytic pathway consists of 10 steps, including three irreversible steps: HK, PFK, and PKM 1/2‐induced reactions. Targeted inhibition of the main enzymes in glycolytic metabolism can inhibit the growth of tumor cells by inhibiting the glycolytic pathway.^70^ It is certain that various enzymes of the glycolysis pathway also play essential roles in tumor invasion and migration, and the related metabolic enzymes will be further explained later.

#### 3.1.2 TCA cycle pathway: indispensable in tumor cells

Mitochondria are the center of cellular energy metabolism, where the TCA cycle occurs. The TCA cycle is a common metabolic pathway in aerobic organisms, and its main functions are to produce precursors for biological macromolecule synthesis.^71^ In addition, it is also the hub of glucose, amino acid, and lipid metabolism. Therefore, tumor cells are very dependent on the TCA cycle to supply energy and synthesize macromolecules.^72^

Through glycolysis, glucose can generate pyruvate. It enters mitochondria and is converted into acetyl‐CoA by pyruvate dehydrogenase (PDH). Acetyl‐CoA and oxaloacetate (OAA) are transformed to citrate‐by‐citrate synthase (CS). Isocitrate is then converted to α‐ketoglutarate (α‐KG) by IDH. α‐KG dehydrogenase catalyzes α‐KG to succinyl‐CoA. After that, succinate is catalyzed by succinate dehydrogenase (SDH) to fumarate, and fumarate hydratase (FH) is catalyzed to produce malate. MDH can catalyze malate to form OAA, and OAA can be reduced to phosphoenolpyruvate (PEP) by phosphoenolpyruvate carboxykinase 2.^73^

Apparently, a variety of enzymes are involved in the TCA cycle, and these enzymes can affect the TCA cycle through the effect of metabolic reprogramming, among which SDH, FH, and IDH are noteworthy. Mutations in SDH and IDH can increase ROS production, thereby promoting tumorigenesis and transformation. Meanwhile, mutations of FH can lead to accumulation of fumaric acid and increased dependence on glycolysis and glutamine recovery.^74^

#### 3.1.3 Other pathways: PPP and HBP

To maintain abnormal requirements for malignant proliferation of tumors, glycolysis intermediates can also be metabolized by other branched pathways, including the PPP and HBP. PPP is mainly used for nucleotide synthesis and the production of reductive NADPH. Meanwhile, the metabolites of HBP can form glycans for protein glycosylation modification.

##### 3.1.3.1 Pentose phosphate pathway

The PPP is a branch of glycolysis that plays an important role in glucose metabolism and acts as a bridge between glucose metabolism, NADPH production and nucleotide precursor synthesis. It also plays a key role in the elimination of cellular ROS and reductive biosynthesis by providing R5P and NADPH.^75^ Rapid division and proliferation of tumor cells requires the synthesis of a large number of nucleotides and lipids, and approximately 85% of the pentose required for DNA synthesis is supplied by PPP.^76^ Therefore, maintaining a certain flow rate of PPP is crucial for tumor cells.

When glucose enters the cell, it is first phosphorylated by HK to form G6P. G6P can produce ATP and other intermediate metabolites through the glycolytic pathway and can also be metabolized through the PPP. When G6P enters the PPP, it can be dehydrogenated by G6PD to produce 6‐phosphogluconic acid (6PG). 6PG is catalyzed by 6‐phosphogluconate dehydrogenase (6PGD) to R5P. NADP+ acts as an electron acceptor in the oxygenation stage of the PPP pathway to receive hydrogen ions and generate NADPH, which provides power for cells to remove ROS.^77^ R5P can be converted to glyceraldehyde 3‐phosphate (G3P) and fructose 6‐phosphate (F6P) by transketolase (TKT). F6P can generate G6P through gluconeogenesis. G6PD and 6PGD are two important oxidoreductases in the PPP. Studies on the regulation of the PPP metabolic pathway mainly focus on the regulation of these two enzymes.

Metabolic reprogramming significantly increased the expression of G6PD, RPIA, and TKT, which proves the presence of high‐flow PPP in human cancer cells. In addition, cancer cells can reduce glycolytic flow and enhance the use of glucose in the PPP to produce more NADPH for antioxidant defense under oxidative stress.^78^ Therefore, tumor cells can control the flow of glucose into glycolysis and the PPP based on the need for NADPH and R5P.

##### 3.1.3.2 Hexosamine biosynthesis pathway

The HBP can mediate the synthesis of the nucleotide sugar UDP‐GlcNAc. It can satisfy the intake demand of various molecules, such as glucose, uridine triphosphate, and acetate, through the regulation of the PPP and AMPK signaling pathways. Therefore, this pathway is recognized as a sensor of bioenergy and metabolism.

In response to metabolic reprogramming of tumor cells, increased uptake of glutamine and glucose can synergistically enhance the activities of HBP. Even though glucose involved in the HBP pathway only occupies a small fraction of intracellular glucose, it is still crucial to the survival of tumor cells. The HBP plays an essential role in the posttranscriptional modification of both tumor suppressor genes and oncogenes.^79^ The pathway can maintain the stability of oncogenic proteins or reduce the stability of tumor suppressor proteins and degrade them. The HBP can convert glucose into 6‐phosphate glutamine and ultimately generate UDP‐GlcNAc, which is a vital cell signaling regulator for tumor growth and is associated with the cancer phenotype.^80^ Under the action of O‐linked β‐N‐acetylglucosamine transferase, UDP‐GlcNAc can transform into O‐GlcnAcylated, which can eventually increase O‐GlcNAc in tumor cells. Recent studies have found that O‐GlcNAc is significantly increased in breast cancer, colon cancer and lung cancer and can promote the activities of cancer cells.^81^ The HBP pathway is part of the central hubs in cell metabolism, which links the metabolism of glucose, protein, nucleic acids, and lipids together. Therefore, further studies of the HBP are conducive to better understanding the metabolism of tumor cells.

#### 3.1.4 Relationship between metabolism enzymes and tumors

The activity and protein expression levels of metabolic enzymes in the glycolysis pathway of tumor cells are upregulated significantly, including HK, PFK, and PK. An increasing number of studies have proven that the main enzymes of tumor glucose metabolism are potential targets for tumor treatment, such as GLUT1, HK2, PFKFB3, and PKM2.^66^ Inhibiting the activity of these glucose metabolism enzymes is beneficial for inhibiting tumor growth.^82^

##### 3.1.4.1 Hexokinase

In most cancer tissues, the expression of the HK family, especially HK2, is significantly increased. HK2, a member of the HK family, is the first key rate‐limiting enzyme in glycolysis and can phosphorylate glucose to glucose 6‐phosphate.^83^ The expression level of HK2 is significantly increased in several cancers. The efficiency of glycolysis in cancer cells is significantly faster than that in normal cells, and the amount of ATP glycolysis also increases significantly.^84^ Therefore, the HK2 catalysis of glucose can be guaranteed; thus, the catalytic efficiency of HK2 is improved, and the proliferation of tumor cells is promoted. High expression of HK2 in cancer cells and its complex regulatory network directly promote an increase in the aerobic glycolysis level of cancer cells, which fulfills the energy demand of rapidly proliferating cells. Moreover, cancer cells can also survive in low oxygen, low glucose and acidic microenvironments. Notably, silencing HK2 can significantly diminish the level of aerobic glycolysis and enhance the transformation of cancer cell metabolism from aerobic glycolysis to OXPHOS.^84^, ^85^ In addition, the interaction between HK2 and voltage‐dependent anion channel can further interfere with the binding between the proapoptotic protein Bax and VDAC, which can escape from mitochondria and trigger apoptosis by forming a channel for cytochrome *C*.^86^ In conclusion, HK2 can accelerate the glycolysis rate and inhibit the apoptosis of tumor cells.

##### 3.1.4.2 Pyruvate kinase

PK oversees catalyzing the final step of glycolysis as a rate‐limiting enzyme. It can catalyze the dephosphorylation of PEP in the glycolysis pathway to form pyruvate and produce ATP. Meanwhile, it is also one of the rate‐limiting enzymes in the glycolysis pathway. Four subtypes of PK are known, including L, R, M1, and M2. Among them, PKM2 is a signature isoenzyme in vigorous nucleic acid synthesis cells.^87^ This enzyme is highly expressed in lung cancer, liver cancer, glioma, and other cancer tissues and is closely linked to staging and prognosis.^88^ Studies have found that PKM2 can be activated through the EGFR pathway and enter the nucleus. Then, PKM2 can bind with phosphorylated β‐catenin protein and activate the transcription of cyclin D1.^89^ Therefore, cell proliferation and tumor formation are promoted.^90^ It links tumor cell cycle regulation with its own energy metabolism through a nonmetabolic mechanism. Multiple studies have shown that reducing PKM2 expression can lead to the death of cancer cells, a reduction in metabolic activity and tumorigenesis. Meanwhile, the sensitivity of tumor cells to docetaxel, cisplatin, and other drugs is improved simultaneously, thereby promoting tumor tissue death.

##### 3.1.4.3 PFK2/fructose 2,6‐bisphosphatase

PFK is also one of the rate‐limiting enzymes in glycolysis and can catalyze the reaction of F6P to fructose 1,6‐diphosphate. PFK2/PFKFB, as an allosteric activator of PFK1, is a main regulator of glycolysis. Its isoform PFKFB3 activates PFK1 allosterically, which promotes the synthesis of fructose 2,6‐diphosphate and the tumor glycolysis process. It is highly related to the survival and proliferation of tumor cells and has been found to be highly expressed in a variety of tumors. Due to metabolic reprogramming, the expression of PFK2 increases significantly. PFK2 can catalyze the conversion of F6P to fructose 1,6‐diphosphate and promote the tetramerization of PK, which later transforms into a glycolytic enzyme complex. Ultimately, glycolysis is promoted. In addition, PFK1 and PFKFB3 are related to the biosynthesis of tumor cells.^91^ Overexpression of PFK1 and PFKFB3 can substantially increase fructose 1,6‐diphosphate, which can bypass the PPP and participate in the synthesis of 5‐phosphoribose. Based on the fact that inhibition of PFKFB3 sensitized cells to cisplatin‐induced apoptosis, a potential anticancer chemotherapy strategy targeting PFKFB3 has been proposed,^92^ but further studies are still needed.

##### 3.1.4.4 Glucose transport protein

The glucose transport protein (GLUT) family consists of 13 members, among which GLUT 1–4 are the basic glucose transporters. GLUT 1 is a glucose transporter on the erythrocyte membrane. Expression of the glucose transporter GLUT1 in tumor cells. GLUT 4 is an insulin‐sensitive glucose transport carrier that is expressed in insulin‐sensitive tissues such as fat and muscle tissues.^93^ It maintains glucose levels and plays an essential role in whole‐body glucose balance. Tumors increase glucose uptake by activating or increasing GLUT 4 translocation and expression to meet their oxygen and energy requirements. Therefore, GLUT1 and GLUT4 are vital in the study of tumor glycolysis.

#### 3.1.5 Relationship between signaling pathways of the glycolysis pathway and tumors

##### 3.1.5.1 Serine/threonine kinase

AKT is one of the main regulators of cellular energy metabolism. It can stimulate aerobic glycolysis in tumor cells, which causes those tumor cells to rely heavily on aerobic glycolysis for survival and proliferation. AKT can also enhance glycolysis through a variety of other cellular mechanisms.^94^ First, it can enhance glucose uptake by promoting glucose transporter expression and glucose transporter membrane translocation. In addition, AKT can indirectly activate PFK1, a glycolytic rate‐limiting enzyme, through direct phosphorylation and activation of PFK2. In addition, AKT can also activate mTOR and promote the abundance of HIF‐1α, which leads to the expression of HIF‐1α‐associated glycolytic genes, such as glucose transporters, HKII, and LDH.^95^

##### 3.1.5.2 Mammalian target of rapamycin

mTOR is a highly conserved serine/threonine protein kinase that is activated in response to nutrients, growth factors, and cellular energy. Dysregulation of mTOR has been implicated in many diseases, including cancer, metabolic diseases, neurological disorders and inflammation.^96^ Over the past decade, mTOR kinase has emerged as a key cellular energy sensor. This is because of its ability to combine different composition processes together, including the composition of nutrients, growth factors, hormone signaling, and available oxygen with proteins and lipids and lysosomal biosynthesis.^97^

mTOR is composed of two different complexes, called mTORC1 and mTOR complex 2 (mTORC2). mTORC2 induces glucose transport by favoring glucose transporter exocytosis (e.g., GLUT4) in muscle. In addition, mTORC2 positively regulates glycolysis in skeletal muscle and liver through the same mechanism that requires AKT activation. Studies have also found that mTORC2 can control aerobic glycolysis in cancer cells (Warburg effect).^98^ In addition, researchers have found that abnormal activation of mTOR induces the production of PKM2 by upregulating a protein that plays a key role in tumorigenesis, thereby promoting aerobic glycolysis in tumor cells.

##### 3.1.5.3 Adenylate‐activated protein kinase

AMPK is a kinase regulated by AMP (adenylate‐activated protein), which is highly conserved in mammals. AMPK can promote ATP production and regulate ATP consumption and therefore is known as a “metabolic and energy receptor.” As a metabolic sensor, AMPK is involved in maintaining energy homeostasis by phosphorylating and regulating proteins that participate in many physiological processes. AMPK can inhibit energy consumption processes and promote energy production pathways to maintain energy homeostasis and ensure the survival of cells under stress conditions. Taking ACC as an example, AMPK can phosphorylate ACC and restrain its activity, decreasing malonyl‐CoA production and reducing fatty acid synthesis. Similarly, the metabolic functions of AMPK in the glycolysis pathway, the TCA cycle, and other carbohydrate metabolism pathways have been widely studied. It has also been found that AMPK plays an important role in glycolysis by regulating PFK and other enzymes that promote glycolysis in cancer cells and uptake glucose by increasing the expression of GLUT.^99^

In addition, AMPK has been implicated as a tumor suppressor because it can control the cell cycle promoter and regulate epigenetic regulators, which can intercept the cell cycle. The serine/threonine protein kinase B‐Raf (BRAF) is phosphorylated by AMPK at Ser729, which changes its binding partner from Ras kinase inhibitor (KSR1) to 14‐3‐3 protein, thereby blocking cell cycle progression and cell proliferation. Likewise, AMPK can disrupt the cell cycle by inducing the expression of p27, p21, p53, and pRb proteins, which play an essential role in regulating cell growth and division. It is obvious that the AMPK signaling pathway plays an indispensable role in tumor glucose metabolism.^100^

#### 3.1.6 Metabolism: a central role in the cancer cell metabolic signaling pathway

Even under restricted energy resources and other adverse circumstances, tumors show the ability to reprogram metabolism to promote cell growth and proliferation. Studies have shown that NAD+/NADH and NADP+/NADPH ratios are higher in tumors than in nontumors, which implies that NAD+ plays an important role in metabolic conversion.^101^ Therefore, tumors prefer the glycolytic pathway rather than OXPHOS even under fully functional mitochondria and normal oxygen conditions.

Glucose uptake by cancer cells is significantly increased, which provides sufficient energy for cancer cells to support their uncontrolled proliferation. As both the GAPDH conversion and lactate production steps depend on NAD+, its level must be increased to meet the requirement of high glycolysis. In addition, the secretion of lactic acid can acidify the TME, which contributes to the invasiveness of tumors. Therefore, NAD metabolism is believed to indirectly affect the growth and invasiveness of tumors.

Moreover, some glycolytic intermediates are also involved in other metabolic pathways that are closely related to NAD metabolism. As in the PPP pathway mentioned earlier, the first step of this pathway is NADP+ dependent and is the main source of NADPH in cells. As a vital cofactor for various antioxidant proteins, NADPH helps combat oxidative stress resulting from accelerated metabolism, induced hypoxia, and accumulation of DNA damage in cancer cells. Apart from that, the serine synthesis pathway (SSP) is also dependent on NAD+. The glycolytic intermediate 3‐PG bypasses glycolysis to produce serine by using NAD+ as a redox cofactor.^102^

NAD+ is not only essential in energy metabolism but also involved in important cell signal transduction processes, which is an important bridge between the two. As an essential component in cancer cell metabolism, NAD+ is involved in deacetylation reactions carried out by deacetylase enzymes such as sirtuins (SIRTs) and polyADP ribose polymerases (PARPs) and generates calcium ions to mobilize molecular cyclic ADP nucleic acid sugar and NAADP. These signaling events are involved in cell cycle progression, transcriptional regulation and DNA repair and have therefore been identified as potential targets for tumor treatment.^103^

It can be concluded that NAD+ plays a central role in the tumor cell metabolic signaling pathway; therefore, NAD+‐dependent processes are highly promising targets for therapeutic applications to treat cancer.

### 3.2 Amino acid metabolism: one of the focuses of modern cancer therapies

Amino acids play important roles in tumor cell growth and survival, including providing carbon sources for TCA, nitrogen sources for base synthesis, and regulating the redox balance. Similar to glucose, energy metabolism and biosynthesis of tumor cells both require the participation of amino acids, and therefore, targeting amino acid metabolism has become one of the focuses of modern cancer therapies.

#### 3.2.1 Glutamine metabolism

**FIGURE 2:**
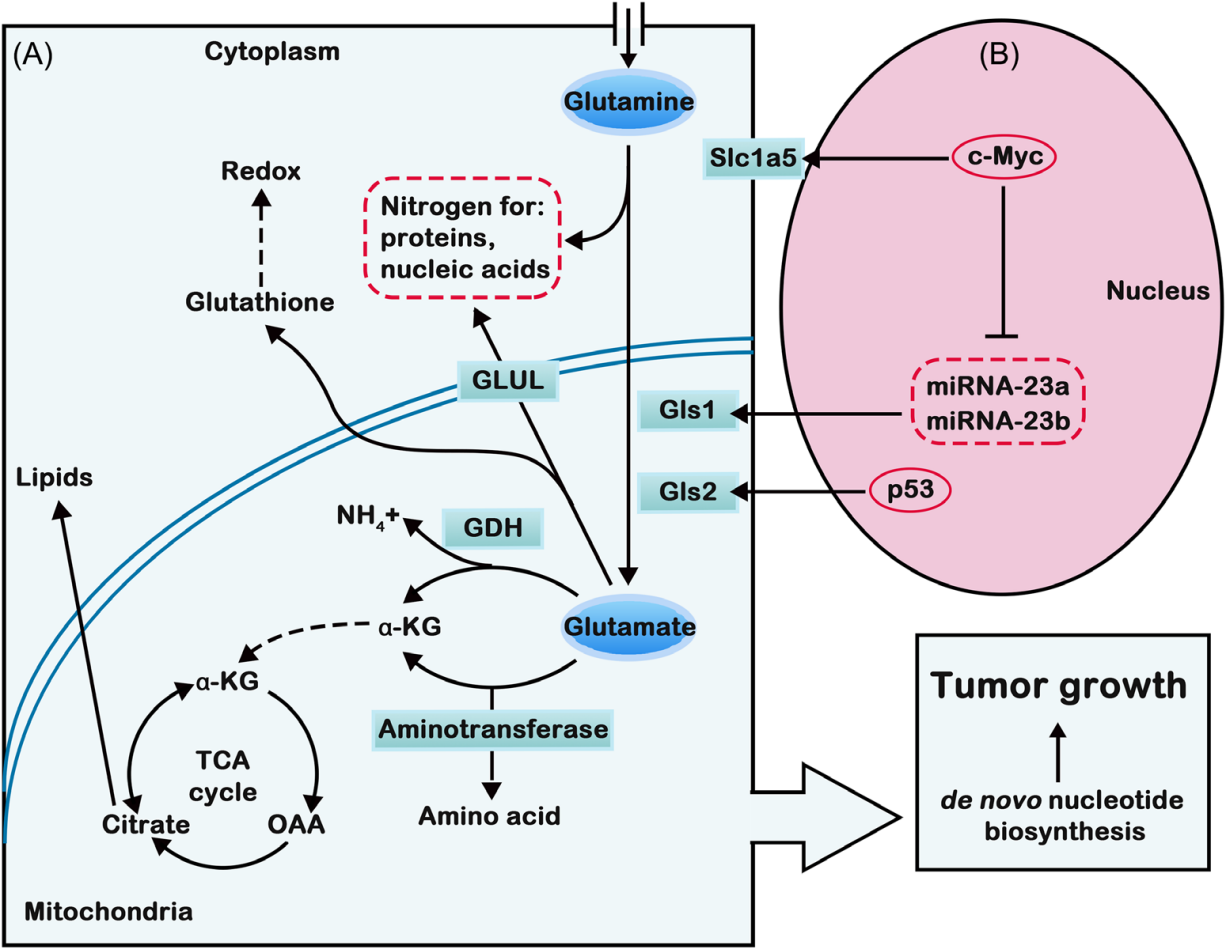
Glutamine metabolic reprogramming in cancer. (A) When glutamine enters tumor cells, it is converted to glutamate, which is catalyzed to form α‐ketoglutarate in mitochondria and enters the tricarboxylic acid cycle (TCA). Meanwhile, this process provides nitrogen for proteins and nucleic acids. When energy and precursors for synthesis are provided, tumor growth is enhanced. (B) The stimulation of the conversion of glutamine to glutamate is due to the upregulation of Slc1a5 and Gls1 by the oncogene c‐MYC. C‐MYC upregulates Gls1 by inhibiting miRNA‐23a and miRNA‐23b. In addition, the downregulation of Gls2 by the tumor suppressive gene p53 can suppress tumor growth.

For decades, researchers have found that glutamine metabolism is crucial for tumor cell growth and proliferation by supporting biomacromolecule synthesis and maintaining bioenergetics and redox homeostasis. Glutamine is the second key source of energy for tumor cells, so it is closely connected with the occurrence and development of tumors. The utilization of glutamine by tumor cells is significantly increased. Glutamine metabolism can promote tumor cell proliferation and inhibit cell death, thereby promoting tumor growth.^104^ Glutamine is the most ingested amino acid in the body and is involved in a series of biological reactions, including energy generation, macromolecule synthesis and signal transduction. The most important role of glutamine is to provide intermediate products for the TCA cycle. Through the transporter solute carrier 1A (SLC1A5), glutamine enters the cell and is then converted to glutamate in response to GLS, GDH, and other enzymes. Glutamate is catalyzed to form α‐KG in mitochondria and enters the TCA cycle to provide energy and synthesize precursors for tumors (Figure 2).^105^

Glutamine also provides a nitrogen source for nucleotide synthesis of purines and pyrimidines and participates in the synthesis of hexosamine and nonessential amino acids (NEAAs). Glutamine is an indispensable factor for promoting tumor cell proliferation because it can provide raw materials for the synthesis of biological macromolecules. As the amino acid demand of cancer cells increases, glutamine always depends on the exogenous supply, which is known as the “glutamine addiction” phenomenon.^106^ The proliferation of cancer cells consumes oxygen, which leads to hypoxia in the TME. To meet its energy requirements, glutamine is one of the main carbon sources.^107^

The effects of glutamine metabolism on tumors are closely related to intracellular signaling pathways. Many studies have shown that glutamine is required for the activation of mTORC1. Inhibition of the SLC1A5 transporter reduces the uptake of glutamine by cells, which is unable to activate mTORC1. Enhanced glutamine metabolism in tumor cells leads to an increase in α‐KG production, which can promote the translocation and activation of mTORC1 to lysosomes. Therefore, the growth and proliferation of tumor cells can be promoted, and the autophagy process can be inhibited. The regulatory relationships between mTORC1 and glutamine metabolism in tumor cells are complicated. The AMPK pathway acts as a metabolic regulator in tumor cells to maintain intracellular energy homeostasis, activate autophagy, and inhibit adipose synthesis and mTORC1 activation.^108^

Moreover, glutamine metabolism can affect the occurrence and development of tumors by affecting autophagy. Autophagy‐associated protein and its upstream signaling molecules can regulate autophagy. Among them, mTORC1 can affect autophagy by regulating metabolism and the cell environment. The α‐KG produced by glutamine metabolism promotes the translocation of mTORC1 to the lysosomal surface by activating RRAG in the presence of EGLNs and activates mTORC1 in the presence of RHEB, which then induces the phosphorylation of ULK1 and TFEB and inhibits autophagy.^109^

Tumor cells take up many glutamines to maintain mitochondrial metabolism. It is not only an important source of nitrogen and carbon but also a regulator of intracellular redox homeostasis and signal transduction. Glutamine plays an important role in cell function, growth and proliferation. Although glutamine metabolism has been investigated for years, its complex metabolic regulatory mechanism still needs to be further explored to prove that glutamine metabolism is a potential target for cancer diagnosis and treatment.

#### 3.2.2 Serine synthesis pathway

Serine is extremely important for the proliferation of tumor cells, and some of its metabolites or key enzymes can regulate the growth of tumor cells. When serine supplementation is insufficient, tumor cells can produce endogenous serine through the SSP.^110^, ^111^

In cancer cells, phosphoglycerate triphosphate, a product of glycolysis, can generate phosphopyruvate by having phosphoglycerate dehydrogenase (PHGDH) and NADPH participate in the reaction process. Then, serine is generated through a series of reactions. PKM2 is expressed during tumor proliferation, and its activity is affected by serine. When the number of serine residues drops, PKM2 is less active. Pyruvate produced by glycolysis is reduced, and therefore, the conversion of 3‐phosphoglycerate (3‐PG) to serine is enhanced.^112^ In addition, the extensive upregulation of glycerophosphatase mutase 1 in tumors can catalyze 2‐phosphoglycerate (2‐PG) production from 3‐PG, which can activate PHGDH to convert 3‐PG from glycolysis to serine synthesis. Studies have shown that the expression of PHGDH is elevated in some patients with breast cancer, pancreatic cancer and lung cancer, and most patients with elevated PHGDH expression show poor prognosis.

Although serine is an NEAA, it plays an essential role in metabolism. One‐carbon units of tumors are mainly derived from serine. Tumor cells can increase the supply of one‐carbon units by rapidly taking in exogenous serine. When exogenous serine is lacking, tumor cells can activate SSP to generate serine. These two pathways allow tumor cells to acquire enough serine to maintain their maximum growth rate. In most rapidly proliferating cells, serine is mainly catalyzed by SHMT2 to produce one carbon unit. It enters the cytoplasm in the form of formic acid to participate in nucleotide synthesis.

#### 3.2.3 Branched chain amino acid metabolism

Amino acids such as leucine, isoleucine and valine are known as branched chain amino acids (BCAAs) due to the branched methyl groups in their hydrophobic side chains. As essential amino acids for the body, they can be directly involved in the composition of tissue proteins. In addition, branched‐chain ketoacids and glutamate can branch out through the metabolism of BCAA aminotransferase (BCAT).^113^, ^114^ Branched‐chain ketoacids can be further oxidized and decomposed to provide a carbon source for TCA and a nitrogen source for nucleotide synthesis. In addition, it can also be involved in epigenetic regulation or excreted from cells and taken up by other tissue cells. Therefore, BCAA metabolism is essential to the activities of the body.

It is believed that BCAA catabolism plays a major role in human cancer. Studies have shown that low BCAA catabolism contributes to cancer progression, and changes in BCAA levels in cancer patients have been a concern for a long period of time.^115^ Elevated BCAA levels in plasma and tumor tissues with decreased BCAA catabolism are observed in a variety of human cancers, including hepatocellular carcinoma, breast cancer, leukemia, early‐stage PDAC, and renal clear cell carcinoma. BCAT and BCKDH, BCAA catabolic enzymes, are closely related to tumor progression. BCAT has two heterogeneous types, which are derived from their respective coding genes and exist in the cytoplasm and mitochondria. BCAT, the enzyme in the first step of BCAA degradation, is upregulated in cancer. BCAT catalyzes the reamination of BCKA in the blood circulation, which enables cancer cells to accumulate BCAAs. The expression of BCAT is particularly important because it controls the intracellular balance between α‐kg and glutamate.^116^ In different types of cancer cells, the increase in BCAT levels enhances the degradation of BCAAs, which leads to a significant reduction in α‐KG. Low α‐KG levels attenuate the activity of EGLN prolyl hydroxylase. EGLN prolyl hydroxylase can block HIF‐1 activation by labeling the proteasomal degradation of HIF‐1. Therefore, reducing α‐KG levels can impair this group of enzymes and lead to HIF‐1 activation. With the activation of HIF‐1, cancer cells can survive in hypoxic conditions.^117^ Meanwhile, BCAA catabolism can provide some precursors for the synthesis of the basic skeleton, such as glutamate, thereby promoting tumor cell proliferation. The first step in BCAA catabolism is to produce glutamates, and these BCAA‐derived glutamates can maintain the amino acid pool for protein synthesis and the nucleotide pool for DNA synthesis. In addition, metabolic abnormalities of BCAAs in tumors are also closely related to the mTOR signaling pathway. Leucine The binding between leucine and its sensor Sestrin 2 protein can activate mTORC1.^118^ mTORC1 can trigger a series of signaling pathways to regulate autophagy and the synthesis of lipids, nucleic acids, and proteins by phosphorylating its downstream effectors.^119^ Genetic alterations or changes in upstream signaling levels can lead to abnormal activity of the mTOR signaling pathway, which may indicate tumor progression or become a new target for cancer treatment.

Metabolic reprogramming of BCAAs changes the levels of these essential metabolites. These metabolites provide nutrients to tumors and build basic substances of the cytoskeleton. The signaling pathways are activated, and the epigenetic profile is reshaped. Thus, the drug resistance of tumors is enhanced, ultimately leading to the survival and rapid invasion of cancer cells. Based on the importance of BCAA metabolic reprogramming in tumor progression, it is believed that enzymes involved in BCAA metabolism may be potential targets for future cancer therapy.

#### 3.2.4 Other NEAAs

##### 3.2.4.1 Aspartate

Aspartate (Asp) is an NEAA that can be synthesized to replenish intracellular stores and degraded from asparagine. Maintenance of asparagine and Asp homeostasis is important to the growth of tumors. In tumor cells, Asp and asparagine not only participate in the proliferation of tumor cells but also regulate signal transduction in cancer cells.^120^ Asparagine and aspartic acid can directly bind to the kinase LKB1, which can affect the kinase activity of LKB1 and the downstream AMPK signaling pathway. The binding of asparagine and LKB1 can inhibit the activity of LKB1, while Asp can enhance the kinase activity of LKB1 to some extent. Therefore, tumor cells can control the activity of the LKB1–AMPK signaling pathway by regulating asparagine metabolism, which affects the survival and proliferation of tumor cells.^121^ Aspartic acid is crucial for cell DNA synthesis and energy supply. Inhibition of intracellular aspartic acid synthesis and extracellular aspartic acid supplementation alone could not restore the proliferation of tumor cells. Aspartic acid limits tumor cell growth by controlling nucleotide and protein synthesis under hypoxia. Therefore, pathways related to aspartic acid availability are a new possible target for cancer therapy in the future.^122^

##### 3.2.4.2 Asparagine

Asparagine (Asn) is an important regulator of amino acid homeostasis, anabolism, and proliferation in tumor cells. Asparagine takes aspartic acid as the substrate and uses glutamine to form the amino group produced by the conversion of ammonia during glutamic acid formation. Under the action of asparagine synthetase (ASNS), asparagine is formed by combining ATP. Intracellular asparagine is known as an amino acid exchange factor that can balance other extracellular amino acids in cells, including glycine, histidine, threonine, and serine.^123^ Research has found that the expression of ASNS in vivo affects the occurrence and development of cancer. For glutamine deficiency, exogenous supplementation of ASN can maintain protein translation and promote the growth of tumor cells.^124^ KRAS can induce the ATF4 pathway during nutrient depletion so that the expression of ASNS can be upregulated. This action contributes to the inhibition of apoptosis, protein biosynthesis and mTORC1 activation, thereby maintaining cell proliferation and alleviating ATF4‐mediated apoptosis. ASNS is dependent on PI3K–Akt regulation. AKT inhibits the KRAS–ATF4–ASNS pathway, coupled with depletion of extracellular ASN.^125^ In endothelial cells lacking glutamine, ASN is essential for restoring protein synthesis, inhibiting ER stress, and reactivating mTOR signaling.

##### 3.2.4.3 Arginine

As a multifunctional amino acid, arginine has a variety of biological functions in metabolism and signaling pathways. Its quantity in cancer cells and related metabolic pathways have an important impact on the growth of tumor cells. Arginine participates in the protein synthesis of tumor cells and is also a precursor for the synthesis of several molecules, including urea, nitric oxide, polyamines, proline, and agmatine.^126^ Arginine is a semi‐essential amino acid that is first formed by citrulline in response to arginine succinate synthetase (ASS) in the body and then by arginine succinate catabolase to arginine and succinate (as part of the urea cycle). ASS is the rate‐limiting step of arginine synthesis, and its expression level is low in most types of cancer, showing heterologous arginine dependence. Therefore, it is believed that ASS is an essential target for cancer treatment.^127^

##### 3.2.4.4 Proline

Proline is important in tumor metabolic reprogramming, which can affect the occurrence and development of cancer. Proline metabolism is associated with ATP production, protein and nucleotide synthesis, and redox homeostasis in tumor cells. Cell clonogenesis and tumorigenesis are inhibited by proline starvation or inhibition of proline biosynthesis. Meanwhile, some cancer cells that depend on exogenous proline showed hyperactivation of the 4E‐binding protein 1 (4EBP1, a cell signaling protein) signal axis, which is mediated by mTORC1. Supplementation with exogenous proline can alleviate endoplasmic reticulum (ER) stress and promote cell homeostasis and clone formation.^128^ Among the proline metabolizing enzymes, pyrroline‐5‐carboxylatereductase is highly expressed in different types of cancer and can promote tumor growth. Due to the significant role of proline in cancer metabolism, inhibition of proline biosynthesis can inhibit tumor growth. Studies have found that overexpression of proline oxidase (POX) in tumor cells can induce apoptosis of tumor cells by increasing the expression of ROS, which may be related to the redox status of tumor cells during proline metabolism.^129^ Similarly, POX maintains the survival of tumor cells by producing ROS to mediate autophagy signals under hypoxic conditions, which further indicates that proline metabolism is a potential tumor therapeutic target.

##### 3.2.4.5 Cysteine

Cysteine is a class of semi‐essential amino acids that are involved in protein synthesis. Cysteine and cystine in tumor cells not only provide energy for tumor cell proliferation but also mediate tumor growth by regulating tumor cell signaling. Apart from being involved in protein synthesis, cysteine is also the rate‐limiting precursor of intracellular GSH synthesis, thus affecting cellular redox homeostasis. Cysteine is unstable in vitro, and the two cysteines are easily oxidized to form cysteines containing disulfide bonds. Most cancer cells mainly take up extracellular cystine through the glutamate/cystine transporter system. Cystine can be rapidly reduced to cysteine in the cell. Some of the cysteines are involved in the synthesis of GSH, and the other part is oxidized out of the cell as cysteine to re‐participate in the system circulation. In the process of cysteine metabolism, cysteine dioxygenase is associated with drug resistance and poor prognosis of tumors. At the same time, cystathionine‐beta‐synthase in the process of its metabolism has also been proven to be related to the proliferation, migration and spread of malignant tumors.^130^

##### 3.2.4.6 Alanine

Alanine is synthesized by alanine aminotransferase and is associated with the central hub of carbon metabolism. It is believed that alanine metabolism is important in cancer. Its biosynthesis is associated with proliferation, which implies that it is important in the metabolism of proliferating cells.^131^ In pancreatic cancer, alanine is considered to be an essential survival signal. Stromal cells secrete alanine for the TCA cycle, thereby promoting the proliferation and survival of pancreatic cancer cells.^132^ Although this evidence is highly linked to the metabolic pathways of cancer, the role of alanine in cancer when compared with other NEAAs is still unclear. Therefore, further research is needed in the future to determine the mechanism of alanine in cancer.

### 3.3 Lipid metabolism: a distinguishing metabolic feature of tumor cells

Enhancement of fatty acid synthesis is another important metabolic feature of tumors that is closely related to glycolysis metabolism.^133^, ^134^ Fatty acids are important lipid molecules in cells and are involved in a variety of biological processes in tumor cells. Due to lipid metabolism reprogramming, tumor cells become increasingly dependent on de novo fatty acid synthesis and exogenous fatty acid uptake so that they can maintain rapid proliferation and provide necessary energy sources under metabolic stress. Compared with normal cells, 90% of fat synthesis in tumor cells comes from de novo synthesis, which can be decomposed into energy through fatty acid β oxidation for the survival of tumor cells under energy stress.^135^

Regardless of the level of extracellular fatty acids in tumor cells, most tumor cells can synthesize fatty acids at a high rate. Some researchers found that almost all esterified fatty acids in tumors came from de novo synthesis.^136^ In contrast, the de novo synthesis level of fatty acids in normal cells is very low. Therefore, de novo synthesis of fatty acids is an important metabolic feature of tumor cells that distinguishes them from normal cells. It is of great significance to explore its specific process and regulatory mechanism as well as its role in the occurrence and development of tumors for a more comprehensive understanding of tumor pathogenesis and treatment.

#### 3.3.1 De novo synthesis

**FIGURE 3:**
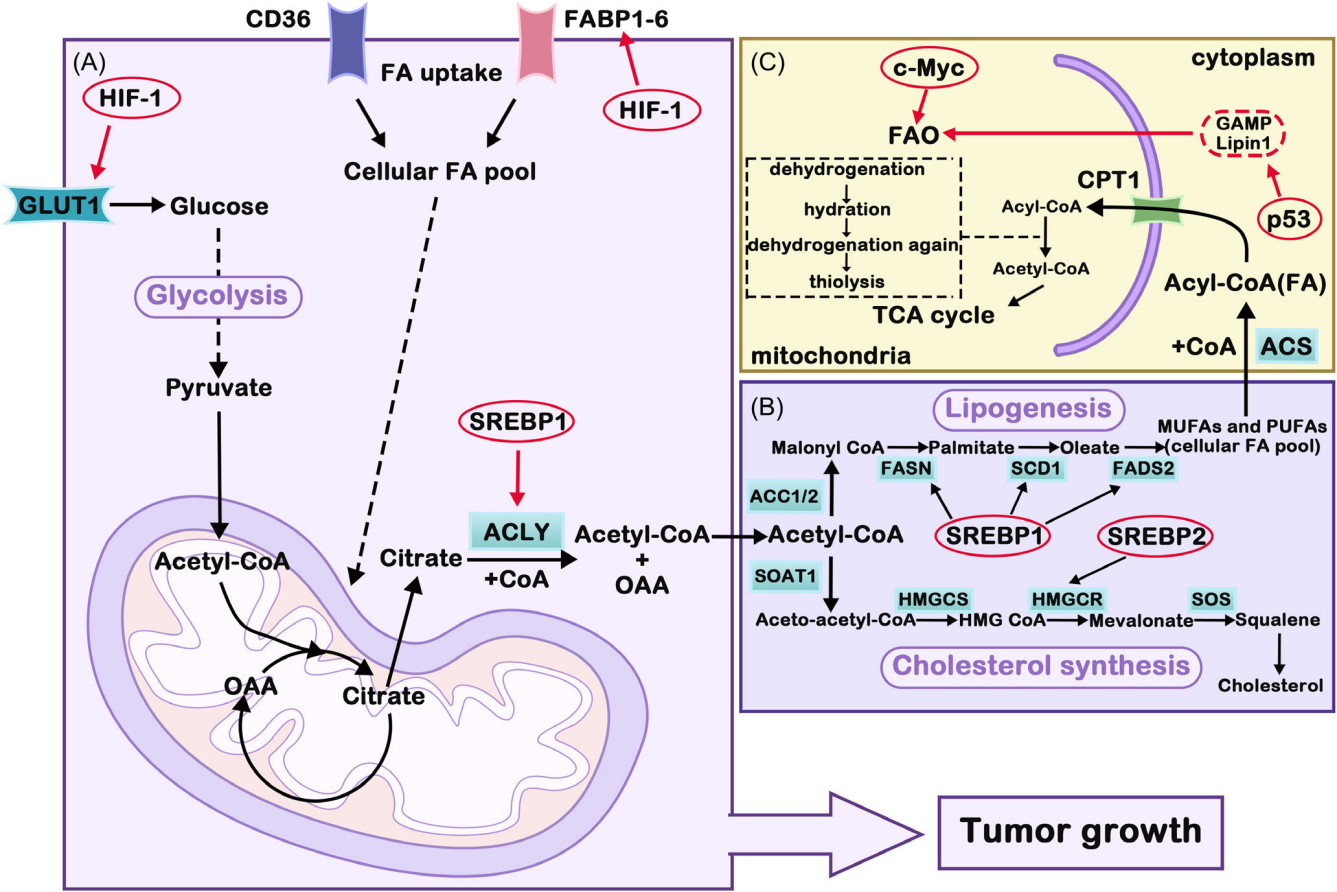
Lipid metabolic reprogramming in cancer. (A) There are two main sources of fatty acids (FAs): exogenous intake and endogenous synthesis (de novo synthesis of FAs). Tumor cells can take up FA through several transporters, including CD36 and FA binding proteins 1–6 (FABP1‐6). These FAs take part in forming the cellular FA pool and participate in forming lipids. For de novo synthesis, the continuous activation of glycolysis produces a large amount of pyruvate, which enters the mitochondria and is catalyzed by pyruvate dehydrogenase (PDH) to produce acetyl‐CoA. OAA produces citric acid under the action of citrate synthase (CS). Then, some of the citric acid enters the tricarboxylic acid (TCA) cycle, while others can cross the mitochondrial membrane and enter the cytoplasm. Acetyl‐CoA in the cytoplasm is an important precursor for fatty acid synthesis. (B) The activation of SREBP upregulates lipogenesis and cholesterol synthesis by expressing genes. Acetyl‐CoA in the cytoplasm participates in lipogenesis and cholesterol synthesis. (C) C‐MYC can upregulate fatty acid oxidation (FAO), which can catalyze FA and be used as an alternative source of energy for tumor cells. Meanwhile, p53 can also promote the catabolism of FA by activating GAMP and lipin 1.

There are two main sources of fatty acids: exogenous intake and endogenous synthesis (de novo synthesis of fatty acids). Most normal cells obtain FA through exogenous pathways, while FA in tumor cells is usually endogenously synthesized de novo, and those key enzymes in the synthesis pathway can be regulated. After entering the bioactive pool, FA can activate the modification of CoA by fatty acyl‐CoA synthetase. Triglycerides (TGs) or sterol esters (SEs) are generated and stored in lipid droplets (LDs). FAs can be used for many purposes in cells, such as being raw material for the activity of the cell membrane, signaling molecules or oxidating carbon dioxide as an energy source. This indicates that FA plays an important role in tumorigenesis and development. The de novo synthesis of fatty acids in tumor cells occurs in several processes. The continuous activation of glycolysis produces a large amount of pyruvate, which enters the mitochondria and is catalyzed by PDH to produce acetyl‐CoA, followed by acetyl‐CoA and OAA to produce citric acid under the action of CS. Some of the citric acid then enters the TCA cycle, while others can cross the mitochondrial membrane and enter the cytoplasm. Acetyl‐CoA and oxaloacetic acid were catalyzed by ATP‐citrate lyase (ACLY). Acetyl‐CoA is an important precursor for the synthesis of fatty acids in the cytoplasm. ACC catalyzes the formation of malonyl‐CoA from partial acetyl‐CoA. Subsequently, one acetyl‐CoA molecule and seven malonyl‐CoA molecules were condensed continuously to produce palmitic acid under the action of FASN (Figure 3). Palmitic acid is a saturated fatty acid with 16 carbon atoms, which can be extended and desaturated to produce fatty acid molecules of different lengths and saturation.^134^

Metabolic pathways to promote specific lipid accumulation in tumor growth can be upregulated by tumors. For example, several key lipid metabolic enzymes in tumors, such as ACC, FASN, and ACLY, were upregulated. Inhibition of these enzymes involved in FA synthesis can suppress tumor growth and metastasis. ACC is the rate‐limiting enzyme of FA synthesis and is highly expressed in breast and prostate cancers, and inhibition of ACC leads to decreased adipose synthesis and apoptosis.^137^ FASN is a key enzyme in FA anabolism, and FASN expression levels are markedly increased in many tumor cells. ACLY, as a cytosolic enzyme that catalyzes the cleavage of citrate to OAA, is upregulated or activated in a variety of tumors. Inhibition of ACLY activity can significantly inhibit the proliferation of tumor cells. SREBP is a family of membrane‐binding transcription factors in the ER. It plays a vital role in the regulation of lipid metabolism73.^138^ The PI3K/AKT/mTOR signaling pathway plays an important role in lipid synthesis. It promotes the expression of genes related to lipid synthesis by activating SREBP1. Then, it upregulates ACLY and FASN to promote de novo FA synthesis.^139^ FA is catabolized during FAO and is an alternative energy source for tumor cells. It was found that inhibition of FAO in glucose‐deficient melanoma cells resulted in the proliferation of ROS and cell death.^140^ It is further demonstrated that FAO is required for melanoma cells to maintain redox homeostasis, cell survival, progression, and metastasis when exposed to metabolic stress.

Although de novo fatty acid synthesis is also present in normal cells, it is limited to the liver, adipose tissue, and breast during lactation. De novo fatty acid synthesis is an important metabolic feature that distinguishes cancer cells from normal cells. It is of great significance to explore the regulatory mechanism of fatty acid synthesis and its role in the development of tumors for a more comprehensive understanding of the pathogenesis and treatment of tumors.

#### 3.3.2 Relationship between metabolism enzymes and tumors

The enhancement of the de novo fatty acid synthesis pathway is the main manifestation of lipid metabolism reprogramming in tumor cells, which involves a variety of key enzymes, mainly including increased expression of ACLY, ACC, and FASN.

##### 3.3.2.1 ATP‐citrate lyase

ACLY is the first key enzyme in the de novo synthesis of fatty acids, which can catalyze the production of acetyl‐CoA from citric acid and serves as a crucial bridge between glucose metabolism and lipid metabolism in cells. Citric acid enters the cytoplasm from the mitochondria, and ACLY can catalyze it to produce acetyl‐CoA, which is an important raw material for fatty acid synthesis. Therefore, with changes in the energy metabolism of cancer cells, the increase in glucose uptake and the acceleration of glycolytic flux will inevitably lead to an increase in citric acid production and ultimately promote fatty acid synthesis in cells. The expression of ACLY in tumors is greater than that in normal tissue and is closely related to tumor stage and prognosis.^141^ Studies have shown that inhibition or knockdown of ACLY can increase the production of ROS, thereby inhibiting the proliferation of tumor cells and inducing apoptosis,^142^ but the mechanism of ROS content enhancement remains unclear. Therefore, further studies are needed to confirm whether ACLY can be used as an effective antitumor target.

##### 3.3.2.2 Acetyl‐CoA carboxylase

ACC can catalyze acetyl‐CoA to produce malonyl‐CoA, which is upregulated by citric acid and glutamic acid and downregulated by acyl‐coenzyme A (e.g., palmitoyl‐CoA) in cells.^142^ Current studies have indicated that ACC is closely linked to the occurrence, development and poor prognosis of cancers. Interfering with its expression or inhibiting its activity can lead to cycle arrest and apoptosis of tumor cells. As ACC is a key enzyme in the synthesis of fatty acids, its expression and activity in tumors will have an important impact on the occurrence and development of tumors. In the future, intervening in the function and expression of ACC may possibly become a treatment for tumors.

##### 3.3.2.3 Fatty acid synthase

FASN is the third key enzyme in fatty acid synthesis. It catalyzes a continuous condensation reaction to produce palmitate ester by using acetyl‐CoA and malonyl‐CoA as substrates. FASN is the most investigated fatty acid metabolism enzyme in cancer at present. The increase in fatty acid synthesis caused by the increased expression of FASN in cancer has been confirmed by a number of studies,^143^ and this process is closely linked to the malignant phenotype, poor prognosis and chemotherapy resistance of tumors. The regulatory mechanisms of FASN in tumors are complex and diverse. Further exploration of its mechanisms is of great significance to the prevention and treatment of tumors in the future.

### 3.4 Others metabolic systems

#### 3.4.1 Lactic acid metabolism

Lactate has been considered in the past as a biomarker of metabolic waste products produced by cellular glycolysis and malignancy. However, recent studies have found that extratumoral lactate accumulation is associated with a higher incidence` of metastasis and decreased overall survival. In addition, lactate can act as an important signaling molecule affecting surrounding cells and plays an essential role in regulating metabolism.^144^

Tumors tend to produce large amounts of lactic acid during glycolysis. Glucose is absorbed and converted to lactic acid by glycolysis, which is recognized as the Warburg effect. To avoid excessive cytosolic acidification, tumor cells must transport lactate out of the cell via MCTs. Reversible proton/lactate cotransporters MCT1 and MCT4 are the main lactate transporters in tumor tissues and can be regulated by intracellular and extracellular monocarboxylate and proton concentration gradients that are prevalent on both sides of the cell membrane.^145^ MCT1 has a high affinity for lactic acid, and the lactic acid outside the tumor is transported into the cell by MCT1. Lactic acid is catalyzed by lactate dehydrogenase B (LDHB) to pyruvate, which is used by tumor cells through the TCA cycle. MCT4 has a low affinity for lactic acid, and the lactate produced by glycolysis is mainly discharged through MCT4. The expression level of MCTs was significantly enhanced in tumor tissues.^146^ However, lactic acid transferred to the outside of tumor cells by MCT can lead to acidification of the TME. Lower extracellular pH can promote tumor cell migration. Low pH values provide a favorable microenvironment for protease activation, including matrix metalloproteinase (MMP) ‐2 and MMP‐9, urokinase‐type plasminogen activator, cathepsin B, D, and L. These proteases reshape the extracellular matrix and promote cell migration and invasion.

In addition, some scholars have proposed the “reverse Warburg effect”, which occurs in tumor‐associated fibroblasts (TAFs).^147^ Cancer cells induce lactate production from nearby TAFs, and lactate produced by TAFs is transferred to adjacent epithelial cancer cells as a substrate for mitochondrial OXPHOS, providing ATP for tumor growth. Studies have shown that GLUT1, HK2, LDH, carbonic anhydrase IX (CAIX), and MCT4 are overexpressed in activated TAFs. Currently, it is believed that the main mechanism of lactate shuttling between TAFs and cancer cells is that lactate is continuously produced and exported by TAFs, and at the same time, lactate is taken up and oxidized by cancer cells, thus playing a part in the occurrence and development of tumors.^148^

Increased levels of lactate, glycolysis metabolites, and lactate shuttling between TAF and tumor cells promoted cancer cell proliferation and metastasis, suggesting that lactate may be a novel target for cancer therapy.

#### 3.4.2 Nucleic acid metabolism

Inside cells, nucleotides are involved in a number of important biological processes, including RNA production and DNA replication, to ensure protein synthesis at different stages of the cell cycle. Nucleotide synthesis requires intermediate or end products of various metabolic pathways to provide carbon and nitrogen precursors, and the related metabolic enzymes synthesized by nucleotide synthesis are regulated in various ways, including transcriptional regulation, allosteric regulation, and feedback regulation. There are two main approaches to nucleotide synthesis in organisms: de novo synthesis and nucleotide salvage.^149^ However, in tumor cells and other proliferative cells, the de novo nucleotide synthesis pathway is strongly activated to meet the metabolic needs of tumor cells. In addition, studies have found that the expression of hypoxanthine‐guanine phosphoribosyltransferase (HGPRT), the main enzyme of the nucleotide remediation pathway, is 33–35% higher in a variety of cancer tissues than in normal tissues, but the mechanism of its effect on tumors is still unclear.^150^ Thymidine kinase (TK1), another important kinase in pyrimidine salvage, is also highly expressed in tumor cells and regarded as an important tumor marker.

The substrates in nucleotide biosynthesis are derived from carbon and nitrogen precursors provided by glycolysis, PPP, the serine‐glycine pathway, the TCA cycle, and glutamine transaminase reactions, including aspartic acid, glutamine, serine and glycine, and CO2. Therefore, in tumor cells, the regulation of these metabolic pathways often leads to changes in nucleotide metabolic pathways. Taking the change in carbon in metabolism as an example, the change in nucleotide metabolism will lead to a change in nucleotide metabolism, and the mechanism regulating the flow of carbon sources into one‐carbon metabolism is the allosteric activation of PKM2 mediated by serine. In addition, in tumor cells, the decrease or loss of argininosuccinate synthase 1 (ASS1), an important metabolic enzyme in the urea cycle, induces the accumulation of aspartic acid, an important substrate in pyrimidine synthesis, which enhances the activity of carbamoyl‐phosphate synthetase (CAD), leads to an increase in pyrimidine synthesis, and finally promotes the proliferation of tumor cells.

#### 3.4.3 Acetate metabolism

Apart from glucose metabolism, glutamine metabolism, and lipid metabolism, cancer cells can also metabolize exogenous acetate to obtain resources for growth under nutrient‐limited conditions.^151^ Acetate and CoA can synthesize acetyl‐CoA under the catalysis of acetyl‐CoA synthetases (ACSS) 1, ACSS3, and ACSS2 located in mitochondria and cytoplasm or nucleus, respectively, which can participate in energy metabolism.^152^ The expression of ACSS2, as a key enzyme mediating the synthesis of acetyl‐CoA from acetic acid, is associated with the aggressiveness of various types of tumors (hepatocellular carcinoma, malignant glioma, breast cancer and prostate cancer). Acetic acid intake was also significantly decreased in ACSS2‐downregulated tumor cells, indicating that ACSS2 plays an important role in the process of tumor cells using acetic acid to synthesize acetyl‐CoA to maintain tumor growth and tumor energy metabolism.^153^

### 3.5 Relationship between epigenetics and metabolism

**FIGURE 4:**
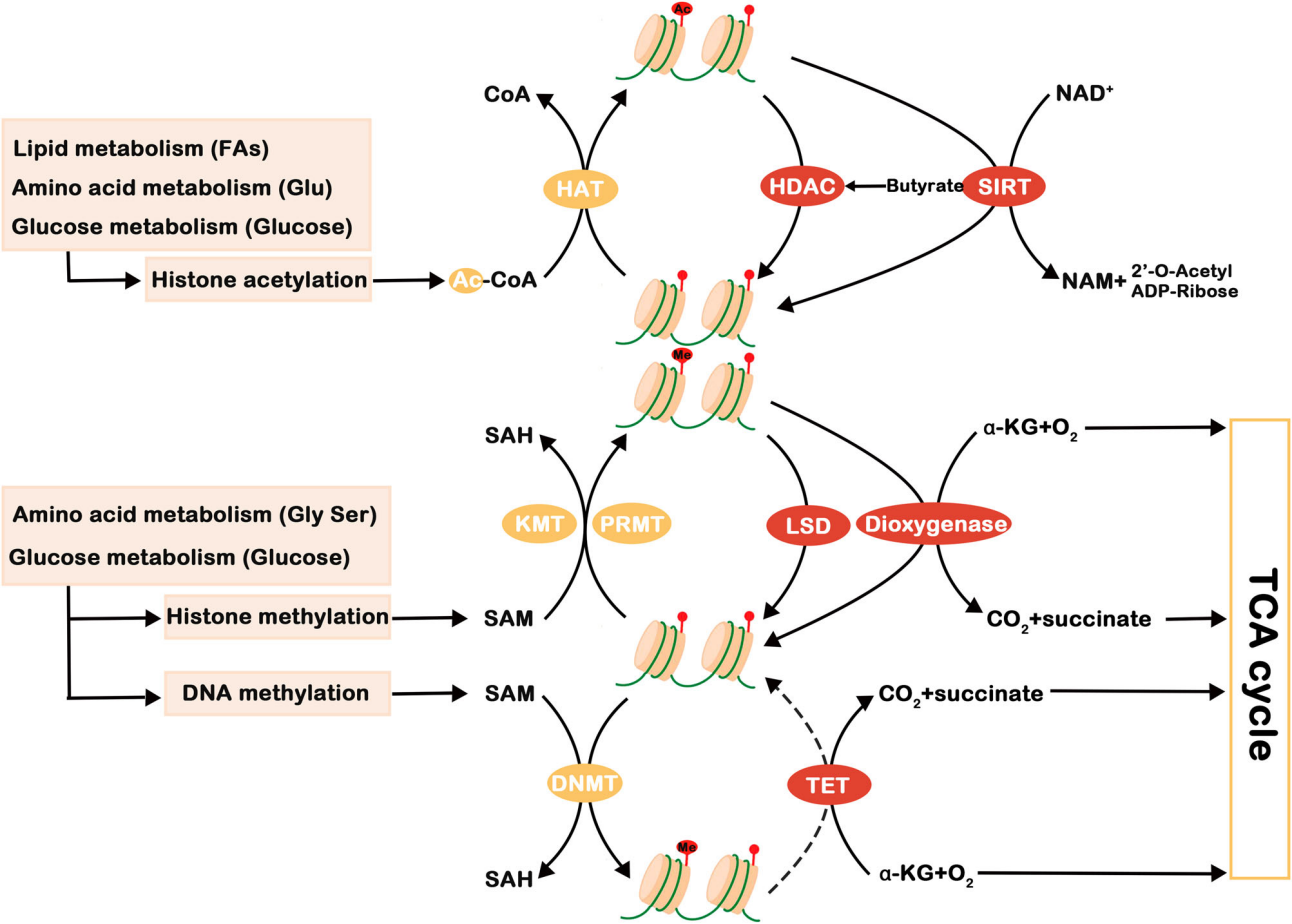
Abnormal methylation or acetylation of histones and DNA is closely related to the occurrence and development of tumors. With the development of detection technology, a more comprehensive understanding of the relationship between metabolic reprogramming and epigenetic modification can be provided. For histone acetylation, histone acetyltransferases (HATs) can shift an acetyl group from acetyl‐CoA to lysine residues, and the reverse reaction is catalyzed by classical or SIRT‐family histone deacetylases (HDACs). For histone/DNA methylation, lysine methyltransferase (KMT) and arginine methyltransferase (PRMT) utilize S‐adenosine homocysteine (SAM) as a methyl donor, whereas amine oxidases lysine demethylases (LSD) 1 and 2 can catalyze the reverse reaction. In the process of DNA methylation, DNA methyltransferase (DNMT) adds a methyl group to CpG dinucleotide cytosine, while the reverse reaction of DNA demethylation is catalyzed by the 10–11 translocation (TET) family of enzymes.

Metabolic reprogramming in tumors induces several changes in metabolism, including abnormalities in metabolites and metabolic enzymes. As a variety of metabolic intermediates are used as substrates during the epigenetic process, changes in tumor metabolic reprogramming can activate or inhibit epigenetic trimming, which affects the expression of epigenetics, such as DNA or histone methylation and histone acetylation (Figure 4). Epigenetics are closely related to the malignant transformation of tumors and can be a new entry point for cancer treatment.

Cellular epigenetics can be influenced by tumor metabolism through at least three different mechanisms. First and foremost, the reprogramming of metabolic pathways leads to altered levels of metabolites, some of which are important cofactors or substrates of key enzymes for epigenetic modification. The second mechanism is related to metabolic products produced after metabolic enzymes are transported into the nucleus. In addition, it is believed that the production of cometabolites can be controlled by regulating the activity of several potent epigenetic enzymes.^154^ The accumulation of metabolites in tumor cells is an important factor that promotes tumor progression.

For histone acetylation, an acetyl group is shifted from acetyl‐CoA to lysine residues by histone acetyltransferases (HATs),^155^ and the reverse reaction is catalyzed by classical or SIRT‐family histone deacetylases (HDACs). For histone/DNA methylation, lysine methyltransferase (KMT) and arginine methyltransferase (PRMT) utilize S‐adenosine homocysteine (SAM) as a methyl donor, whereas amine oxidases lysine demethylases (LSD) 1 and 2 can catalyze the reverse reaction. In the process of DNA methylation, DNA methyltransferase (DNMT) adds a methyl group to CpG dinucleotide cytosine, while the reverse reaction of DNA demethylation is catalyzed by the 10–11 translocation (TET) family of enzymes.^156^ Changes in epigenetic expression are closely associated with malignant transformation of tumors.

With the further study of epigenetics, its role in the development of malignant tumors has been recognized. Therefore, epigenetic therapy based on DNA or histone methylation and histone acetylation is also a hot topic in the future treatment of tumors.

### 3.6 Immune metabolism: interactions between tumor cells and immune cells

Complex metabolic patterns similar to those of tumor cells also exist in immune cells. There are significant differences in the energy consumption patterns of immune cells between the resting state and the activated state. At rest, immature T cells mainly produce ATP through OXPHOS, and when activated into effector T cells, they mainly produce energy through glycolysis. In addition, different immune cells mainly produce energy in different ways, so the differentiation of immune cell subsets can be affected by diverse metabolic patterns.^157^

Interactions between tumor cells and immune cells affect metabolic reprogramming. On the one hand, tumor cells compete to consume nutrients in the immune microenvironment, such as glucose; on the other hand, tumor cells secrete various metabolites, which affect gene expression and signal transduction in tumor cells and reshape the tumor immune microenvironment.^158^ Tumor metabolism can also regulate immune cell metabolism reprogramming by releasing tumor‐derived metabolites, such as lactic acid and prostaglandin E 2. mTOR and AMPK signaling pathways are the key pathways for regulating immune cell metabolism reprogramming, which in turn affects the tumor immune microenvironment. The interaction between tumor cells and immune cells contributes to metabolic competition in the tumor immune microenvironment, which limits the normal metabolism of nutrients and forms an acidic environment, eventually leading to the weakening of the antitumor immune response and the formation of an immunosuppressive microenvironment.^157^

## 4 METABOLIC REPROGRAMMING‐RELATED TREATMENT OPTIONS: DRUGS AND DIET

Surgery, chemotherapy, and radiotherapy have long been the conventional options for cancer treatment. The choice of treatment varies, depending on the type, extent, and progression rate of cancer and the condition and treatment response of the patient. Although the above options are available for cancer therapy, different cancer cells exhibit diverse and multiple unpredictable responses toward different treatments, and cancers will contribute to progress when the current treatments fail. Therefore, it is crucial to explore alternative therapeutic options for cancer.^159^ As research progresses, the mechanism of metabolic reprogramming in cancer has been investigated thoroughly, and it is believed that targets related to metabolic reprogramming are the core to develop new effective therapies to eradicate cancer.

At present, based on the method of intervention, therapeutic approaches are basically divided into two types: nutritional treatments by dietary‐related modulation^159^ and pharmacological treatments by designing anticancer drugs.^160^ With further research, the combination of different treatments is also considered a promising therapeutic approach. Nevertheless, metabolic options do not guarantee the success of metabolic inhibition or suppression, as metabolic reprogramming has its own specific complexities. For example, the plasticity of tumors can have an impact on metabolic complexity. Prostate cancer cells use glycolysis and OXPHOS at different cancer stages of progression to supply energy.^161^ Normal prostate epithelial cells are highly dependent on glycolysis for energy production, while for prostate cancer, cells gradually shift to an OXPHOS‐dependent metabolic pattern in the early stage and prefer to undergo glycolysis as normal during tumor progression. Therefore, it is quite important to refine the process to identify the patients who respond best to a suitable type of therapy. Apart from that, most metabolic targets associated with relevant therapies tend to be overexpressed in cancer, but they vary depending on the individual dependence of metabolic pathways.^162^ Therefore, the key for targeted metabolism is to better understand the specific metabolic dependence in different tumors, thus limiting the growth of relevant tumors. By doing so, more treatments are promising for exploration in the near future, and better cancer treatment options can be provided in the near future.

### 4.1 Anticancer drugs targeting metabolic reprogramming

As research progresses, knowledge and understanding of the multiple targets of metabolic reprogramming is gradually increasing, which has facilitated the development of targeted drug therapies for various aspects of metabolism. Therefore, the targeting of relevant metabolic pathways with specific drugs has the potential to significantly reduce the growth and proliferation of cancer cells. Various drugs classified according to different metabolic pathways, including glucose metabolism, amino acid metabolism, one‐carbon metabolism, lipid metabolism and other metabolic and growth pathways, are summarized in Table 1. While most anticancer drugs are still in the preclinical state, several drugs have already shown great potential in cancer therapy and entered the clinical trial state or even phase IV trials.^163^, ^164^, ^165^ Several drugs, such as orlistat mainly for obesity^166^ and metformin for diabetes,^167^ were not originally developed for the intention of cancer therapy, but they also performed well in targeted cancer treatments coincidentally. Both drugs that have been clinically approved for tumor therapy and drugs that have been clinically widely used with the potential for tumor therapy are summarized in Table 2. As the mechanisms of metabolic reprogramming of cancer cells are further elucidated, current therapeutic approaches may also be improved and updated, and more treatment strategies may be developed.

**TABLE 1:** Multiple anticancer drugs targeting metabolic reprogramming.

Target	Drug	Latest state of development	References
**Glucose metabolism‐related**			
Hxokinase (HK)			
HK1	Lonidamine	Phase III	^168^
HK2	3‐Bromopyruvate (3‐BP)	Preclinical	^169^
	2‐Deoxyglucose (2‐DG)	Phase II	^170^
	Genistein‐27	Preclinical	^171^
	Benserazide	Preclinical	^172^
	Resveratrol	Phase I	^173^
	Astragalin	Preclinical	^174^
	Chrysin	Preclinical	^175^
Pyruvate kinase (PKM)			
PKM2	Shikonin	Preclinical	^176^
	Orlistat	Preclinical	^177^
Fructose‐2,6‐bisphosphatase (PFKFB)			
PFKFB3	3PO/PFK158	Phase I	NCT0204486
	compound 26	Preclinical	^178^
	PQP	Preclinical	^179^
	KAN0438757	Preclinical	^180^
PFKFB4	5MPN	Preclinical	^181^
Glucose transporters (GLUTs)			
GLUT1	WZB115	Preclinical	^182^
	WZB117	Preclinical	^183^
	Fasentin	Preclinical	^184^
	STF‐31	Preclinical	^185^
	Curcumin	Preclinical	^186^
	Cytochalasin B	Preclinical	^187^ ^188^
GLUT2	Phloretin	Preclinical	^189^
GLUT4	Silybin	Phase I	^190^
	Ritonavir	Preclinical	^191^
GLUT5	2,5‐AM	Preclinical	^192^ ^193^
Pyruvate dehydrogenase kinase (PDK)			
pan‐PDK	Dichloroacetate (DCA)	Phase II	^194^
	Mitaplatin (cisplatin and DCA fusion)	Phase I	^195^
PDK1	AR‐12	Phase I	^196^
Glyceraldehyde‐3‐phosphate dehydrogenase enzyme (GAPDH)			
GAPDH	Koningic acid (KA)	Preclinical	^197^
	Iodoacetate	Preclinical	^198^
	3‐Bromopyruvate (3‐BrPA)	Preclinical	^199^
**Lactate metabolism**			
Lactate dehydrogenase (LDHA)			
LDHA	Oxamate	Preclinical	^200^
	Galloflavin	Preclinical	^201^
	FX11	Preclinical	^202^
Monocarboxylate transporter (MCT)			
MCTs	α‐Cyano‐4‐hydroxycinnamic acid (CHC)	Preclinical	^203^
MCT‐1	AR‐C155858	Preclinical	^204^
	AZD3965	Phase I	^205^
**Tricarboxylic acid cycle (TCA)**			
Mutant isocitrate dehydrogenase (IDH)			
Mutant IDH1	Ivosidenib(AG‐120)	Phase IV	^164^
	IDH305	Phase II	^206^
	BAY1436032	Phase I	^207^
	Olutasidenib (FT‐2102)	Phase II	^208^
Mutant IDH2	Enasidenib (AG‐221)	Phase IV	^163^
Mutant IDH1/2	Vorasidenib (AG‐881)	Phase III	^209^
Pyruvate dehydrogenase (PDH)	CPI‐613	Phase III	^210^
**Pentose phosphate pathway (PPP)**			
Glucose‐6‐phosphate dehydrogenase(G6PD)	Polydatin	Preclinical	^211^, ^212^
	Dehydroepiandrosterone (DHEA)	Phase I	
	RRx‐001	Phase II	NCT02452970
6‐Phosphogluconate dehydrogenase (6PGD)	6‐Aminonicotinamide (6‐AN)	Preclinical	^213^
**Amino acid metabolism**			
Glutaminase (GLS)			
GLS1	BPTES	Preclinical	^214^
	CB‐839	Phase II	^215^
	JHU‐083	Preclinical	^216^
	969	Preclinical	^217^
**Solute carrier family (SLC)**			
l‐type amino acid transporter (LAT)^218^/ (SLC7)			
LAT1/SLC7A5	JPH203	Preclinical	^219^
LAT3/SLC43A1	ESK246	Preclinical	^220^
LAT1/3	ESK242	Preclinical	^220^
SLC7A11	Sulfasalazine	Preclinical	^221^
SLC1A5/ASCT2	AABA	Preclinical	^222^
Serine hydroxymethyltransferase (SHMT)			
SHMT1/2	SHMT‐IN‐2	Preclinical	^223^
	Ro07‐7957	Preclinical	^224^
	AGF347	Preclinical	^225^
Phosphoglycerate dehydrogenase (PHGDH)			
PHGDH	NCT‐503	Preclinical	^226^
	CBR‐5884	Preclinical	^227^
l‐asparagine	Bacterial l‐asparaginases	Preclinical	^228^
Arginine	Arginine Deiminase (ADI‐PEG20)	Phase III	^229^
Methionine	Recombinant Methioninase	Preclinical	^230^
**One‐carbon metabolism‐related**			
Methylene tetrahydrofolate dehydrogenase (MTHFD)			
MTHFD2	LY345899	Preclinical	^231^
MTHFD1/2	Carolacton	Preclinical	^232^
Dihydrofolate reductase (DHFR), thymidylate synthase (TS), glycinamide ribonucleotide formyltransferase (GARFT)			
TS, DHFR, GARFT	Pemetrexed/MTA/LY231514	Phase IV	^233^
TS, DHFR	Amethopterin/MTX/methotrexate	Phase IV	^234^, ^235^
TS	Capecitabine/Xeloda	Phase IV	^236^, ^237^
	5‐Fluorouracil/Adrucil	Phase IV	^238^
Phosphoribosyl pyrophosphate amidotransferase (PPAT)	6‐Mercaptopurine	Phase III	^239^, ^240^
	6‐Thioguanine	Phase III	^241^, ^242^
**Lipid metabolism‐related**			
Fatty acid synthase (FASN)^243^			
FASN	Cerulenin	Preclinical	^244^
	C75	Preclinical	^245^
	Epigallocatechin‐3‐gallate (EGCG)	Phase I/II	^246^
	Orlistat	Preclinical	^247^
	C93	Preclinical	^245^, ^248^
	GSK837149A	Preclinical	^249^
	TVB‐2640	Phase II	NCT03179904
	GSK2194069	Preclinical	^250^
	Triclosan	Preclinical	^251^
	Fasnall	Preclinical	^252^
Acetyl‐CoA carboxylase (ACC)			
ACC	Soraphen‐A	Preclinical	^253^
	5‐(Tetradecyloxy)‐2‐furoic acid (TOFA)	Preclinical	^254^
	ND‐646	Preclinical	^255^
	ND‐654	Preclinical	^256^
ATP‐citrate lyase (ACLY)			
ACLY	SB‐204990	Preclinical	^257^, ^258^
Stearoyl‐CoA desaturase (SCD)			
SCD	BZ36	Preclinical	^259^
	A939572	Preclinical	^260^
Sterol regulatory element‐binding protein (SREBP)			
SREBP	Fatostatin	Preclinical	^261^
	Betulin	Preclinical	^262^
**Oxidative phosphorylation (OXPHOS)**			
Complex I	Metformin	Both preclinical and clinical	^263^
	Phenformin	Both preclinical and clinical	^264^
Complex II	Lonidamine	Phase III	^265^
Complex III	Atovaquone	Early Phase I	NCT0464803
Complex IV	Arsenic trioxide	Phase III	NCT0050476
	Nitric oxide	Both preclinical and clinical	^266^
**Other growth pathways‐related**			
mTORC1	Rapamycin	Phase III	^267^
AKT/mTORC1	Halofuginone	Phase II	^268^
PI3K/AKT	Afuresertib	Phase II	^269^
	Uprosertib	Phase II	^270^
	Ipatasertib	Phase III	^271^
KRAS mutation	Sorafenib	Phase II	^272^
	Trametinib	Phase III	^273^
	Selumetinib	Phase III	^274^
HIF1	Echinomycin	Preclinical	^275^
	PX‐478	Phase I	^276^
	2‐Hydroxyestradiol	Preclinical	^277^
	2‐Methoxyestradiol/2ME2/Panzem	Phase II	^278^
AMPK	AICAr/Acadesine	Phase I/II	^279^
	Salicylate	Preclinical	^280^
	A‐769662	Preclinical	^281^
	OSU‐53	Preclinical	^282^

**TABLE 2:** Clinically approved drugs and their therapeutic potential in cancers.

Drug	Target	Cancers	References
Ivosidenib(AG‐120)	Mutant IDH1	Relapsed or refractory acute myeloid leukemia (R/R AML)	^164^
Enasidenib (AG‐221)	Mutant IDH2	R/R AML	^163^
Pemetrexed/MTA/LY231514	TS, DHFR, GARFT	Malignant mesotheliomas, NSCLC	^233^, ^283^
Amethopterin/MTX /methotrexate	TS, DHFR	Acute myeloid leukemia (AML)	^234^
Capecitabine/Xeloda	TS	Advanced breast and colorectal cancers	^236^
5‐Fluorouracil/Adrucil		Colorectal cancer, gastric cancer	^238^
Metformin	Complex I	Solid tumors	^263^, ^284^
Phenformin		Solid tumors	^264^
Nitric oxide	Complex IV	NSCLC	^266^

### 4.2 Nutritional approaches targeting metabolic reprogramming

Metabolic reprogramming facilitates metabolic pathways for multiple metabolites, which then in turn serve the metabolic pathways above, thereby promoting the growth and proliferation of tumors and inhibiting tumor apoptosis. As a result, cancer is also considered to be a metabolic disease, and this view influences the approach to cancer management and prevention.^285^, ^286^ Modulation of dietary nutrients affects cancer in different ways. Dietary compositions influence the utilization of nutrients in the plasma and afterwards in the microenvironment of the body cells, including tumor cells.^287^ The utilization of nutrients in the plasma therefore alters the microenvironment of the body cells, including tumor cells, and eventually alters their metabolic activity, thus affecting many physiological processes such as cell growth and proliferation, metabolic requirements^288^, ^289^ and drug sensitivity.^290^ Diet can also determine signal transduction through nutrient‐sensing pathways that are closely linked to oncogenic signaling.^291^

Based on the increasing understanding of the metabolic mechanism and the nutritional demands of tumors, dietary nutritional modification has therefore been proposed to serve as a complement to conventional cancer therapies.^292^ Recent studies have mainly focused on the influence of macronutrients (carbohydrates, protein/amino acids, and fat) rather than micronutrients as anticancer metabolism therapies, and nutritional interventions are mainly divided into either dietary restriction or supplementation with nutrients.^293^ Various nutritional interventions as cancer therapeutic methods are summarized in Table 3.

**TABLE 3:** Nutritional interventions targeting metabolic reprogramming.

Nutritional interventions	Metabolism	Latest state of development	References
Dietary restriction			
Fasting	–Reduction of IGF‐1 levels mediates differential protection between normal and cancer cells in response to fasting.	Preclinical	^294^
Glucose restriction (Ketogenic diet)	–Dietary glucose, contributes to the initiation of cancer. –Low glucose diet with isocaloric value such as ketogenic diet can reduces glucose levels in the blood. –Elevates ketone bodies, which provide energy to the brain and other tissues and cannot be the energy sources of cancer cells. –Reduces circulating insulin, and lessening the pro‐proliferative activity in the PI3K/mTORC1 pathways.	Clinical trials ongoing	^295^, ^296^, ^297^
Fructose restriction	–Cancer cells import large amount of fructose through GLUT5. – Long‐term consumption of fructose even in moderate doses increase the incidence of colorectal cancer by activating glycolysis in intestinal tumors and inducing fatty acid synthesis and tumors growth.	Preclinical	^298^, ^299^
Amino acid deprivation	–Exogenous reduction of amino acid intake to inhibit tumors growth and proliferation.	Clinical trials ongoing	^300^
Serine, glycine	–Exogenous serine is in high demand in cancer cells, glycine is basically synthesized directly through serine. –Serine deprivation can reduce circulating serine levels, trigger SSP and suppresses glycolysis.	/	^110^, ^301^
Methionine	–Many cancer cell lines are methionine auxotrophs, so they need large amount of exogenous methionine. –Cellular redox stress can be reduced by Dietary methionine reduces, without which cancer cells cannot sustain cytosolic redox homeostasis pathways.	/	^302^
Cysteine	–Indispensable for promoting survival and proliferation. –Increased metabolic demands must be satisfied through extracellular cysteine and de novo cysteine production. –Cystine deprivation can deprive antioxidant GSH, which influences the responding to oxidative damage of the cells.	/	^303^, ^304^
Dietary supplementation			
Mannose	–Interferes with glucose metabolism and inhibits cancer cell growth, for mannose and glucose use the same metabolic enzymes. –Dietary mannose supplementation can disrupt glucose metabolism, thus facilitate cancer cells to be more sensitive to chemotherapy‐induced apoptosis.	Preclinical	^305^
Amino acid supplementation Histidine	–Histidine degradation pathway competes for tetrahydrofolate (THF) with the essential nucleotide‐synthesis enzymes. –Low flux through the histidine degradation pathway can render cells refractory to methotrexate treatment. –Diet‐mediated histidine therefore increase in flux reduces THF availability, de novo nucleotide synthesis and cancer cell survival.	Preclinical	^306^, ^307^

Notably, cancer cells are highly metabolically heterogeneous. Nutritional intervention must be precise, as different cancer cells show significant differences in the metabolism of the same nutrients. Therefore, nutritional intervention requires precision. Additionally, when one metabolic pathway is blocked, cancer cells can usually switch or activate other pathways to escape stress damage. Hence, various metabolic therapies should be integrated for multiple metabolic pathways to improve therapeutic efficacy. Before dietary intervention becomes a common approach for cancer treatment, further preclinical studies and clinical trials are still required to better understand the dietary changes in metabolic reprogramming.

## 5 CONCLUSION

Tumors are a complex metabolic disease. Starting from the understanding of the “Warburg effect,” great progress has been made in the field of cancer metabolism, and research on relevant metabolic pathways is becoming increasingly extensive. With the deepening of tumor metabolism research, the mechanism of tumor generation and development has been further clarified; that is, metabolic reprogramming plays an important role in the growth and proliferation of tumors. Through the interaction of a variety of circulatory and metabolic pathways, as well as the use of alternative substrates, the metabolic plasticity of tumors is endowed. Moreover, tumor cells undergo metabolic regulation to adapt to changes in their growth environment, which can be a target for tumor therapy. For tumor therapy, in addition to traditional nutritional interventions, drug therapies targeting key proteins in related signaling pathways are also expected to become emerging approaches. Therefore, studying the important metabolic pathways and key metabolic enzymes of tumors is of great significance for the diagnosis and treatment of tumors, providing more potential tumor treatment options. The combination of multiple strategies is also considered to be a promising treatment. However, this review only summarized the mechanisms and potential applications of metabolic reprogramming for tumor therapy based on existing studies. More research and technological development are still necessary, and more evidence and further exploration of metabolic changes are needed to formulate targeted anticancer strategies and explore the possibility of more tumor treatments.

## AUTHOR CONTRIBUTION

S. N., X. H., and X. M. selected the topic of the review, performed the literature search, and wrote the manuscript. Y. X., Y. Q., K. S., J. Y., and T. Z. contributed to include literature and helped to edit the final manuscript. K. T. and Y. W. designed the figures and modified the manuscript. All authors read and approved the final version of the manuscript.

## CONFLICT OF INTEREST STATEMENT

There are no conflict of interest to declare.

## ETHICS STATEMENT

This review does not address ethical issues.

## FUNDING INFORMATION

The authors declare that there was no funding for the study.

## Data Availability

Not applicable.
